# Computational textural mapping harmonises sampling variation and reveals multidimensional histopathological fingerprints

**DOI:** 10.1038/s41416-023-02329-4

**Published:** 2023-06-30

**Authors:** Otso Brummer, Petri Pölönen, Satu Mustjoki, Oscar Brück

**Affiliations:** 1 Hematoscope Lab, Helsinki University Hospital, Comprehensive Cancer Center and Center of Diagnostics, Helsinki, Finland; 2 Hematology Research Unit Helsinki, University of Helsinki and Helsinki University Hospital Comprehensive Cancer Center, Helsinki, Finland; 3 Translational Immunology Research Program, University of Helsinki, Helsinki, Finland; 4 Department of Pathology, St. Jude Children’s Research Hospital, Memphis, TN USA; 5 iCAN Digital Precision Cancer Medicine Flagship, Helsinki, Finland; 6 Department of Clinical Chemistry and Hematology, University of Helsinki, Helsinki, Finland

**Keywords:** Image processing, Medical genomics, Renal cell carcinoma

## Abstract

**Background:**

Technical factors can bias H&E digital slides potentially compromising computational histopathology studies. Here, we hypothesised that sample quality and sampling variation can introduce even greater and undocumented technical fallacy.

**Methods:**

Using The Cancer Genome Atlas (TCGA) clear-cell renal cell carcinoma (ccRCC) as a model disease, we annotated ~78,000 image tiles and trained deep learning models to detect histological textures and lymphocyte infiltration at the tumour core and its surrounding margin and correlated these with clinical, immunological, genomic, and transcriptomic profiles.

**Results:**

The models reached 95% validation accuracy for classifying textures and 95% for lymphocyte infiltration enabling reliable profiling of ccRCC samples. We validated the lymphocyte-per-texture distributions in the Helsinki dataset (*n* = 64). Texture analysis indicated constitutive sampling bias by TCGA clinical centres and technically suboptimal samples. We demonstrate how computational texture mapping (CTM) can abrogate these issues by normalising textural variance. CTM-harmonised histopathological architecture resonated with both expected associations and novel molecular fingerprints. For instance, tumour fibrosis associated with histological grade, epithelial-to-mesenchymal transition, low mutation burden and metastasis.

**Conclusions:**

This study highlights texture-based standardisation to resolve technical bias in computational histopathology and understand the molecular basis of tissue architecture. All code, data and models are released as a community resource.

## Background

Histopathological examination of haematoxylin and eosin-stained (H&E) tissue sections remains one of the cornerstones in the diagnostics and prognostics of human cancers. In computational histopathology, image analysis algorithms are trained for detection and classification tasks generally performed by pathologists [1]. Deep learning-based models such as convolutional neural networks (CNNs) and visual transformers have been able to identify both standard histological patterns such as tumour grade [2] and mitosis [3] but also novel patterns related to genomic alterations [4–6], gene expression [7, 8] and viral tumourigenesis [9]. However, no standardisation steps are applied to resolve sampling variations, which are evident even at low magnification, although this could severely bias study results.

Given the promising efficacy of immunotherapy as adjuvant [10] or first-line combination therapy [11], computational histopathology could provide an inexpensive tool to identify patients associated with poor survival or treatment sensitivity such as by quantifying tumour-infiltrating lymphocytes to predict immunotherapy response [12, 13].

With over 10,000 diagnostic slides and associated genomic, transcriptomic, epigenomic, and clinical data from 32 cancer types, The Cancer Genome Atlas (TCGA) is a unique multi-omics archive for biomedical research [14]. Previous reports on ccRCC genetics have illustrated remodelling of cellular metabolism [15], abundant indel mutation [16], and adaptive immunity [17]. TCGA image data have been used to indicate that *PBRM1*-mutated ccRCC samples can be identified from H&E images [7], and that combination of genomic and image data can improve prognostication compared to standard staging [18, 19].

Here, we explored the lymphocyte infiltration and textural architecture in TCGA H&E-stained ccRCC digital sections. Inspired by a recent normalisation technique evaluated on TCGA RNA-seq data [20], we propose computational texture mapping to overcome sampling variation and illustrate its value in harmonising lymphocyte infiltration. Finally, we uncover the intricate network between histopathological architecture and clinical, immunological, genomic, and transcriptomic correlates.

## Methods

### TCGA and Helsinki ccRCC patient cohorts

**Fig. 1:**
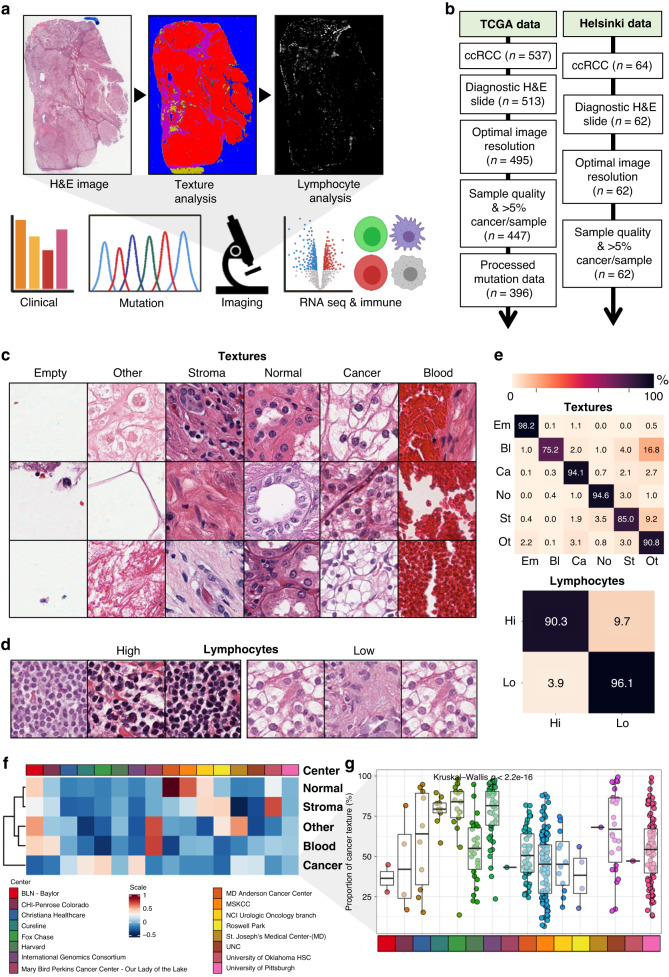
Study design. **a** Digital haematoxylin and eosin (H&E)-stained slides of clear-cell renal cell carcinoma (ccRCC) patients were collected from The Cancer Genome Atlas (TCGA). Six texture subtypes as well as lymphocytes were detected and quantified with convolutional neural networks. Imaging data were integrated with clinical, genomic, transcriptomic and transcriptome-based immune profiling data. **b** Flow chart of the patient number included in this study. **c** Image tile examples annotated by texture subtypes and **d** lymphocytes. **e** Classification accuracy of the computational texture mapping (CTM) and lymphocyte classifiers. **f** Heatmap of median proportion of texture subtypes by participating TCGA clinical site. **g** Cancer tissue proportion by participating TCGA clinical site. The box plots indicate the interquartile ranges and median values.

We collected histology images from TCGA portal (https://portal.gdc.cancer.gov/) and clinical, processed transcriptome data and CIBERSORT-based immune fractions from https://gdc.cancer.gov/about-data/publications/panimmune [21] and https://gdc.cancer.gov/node/905/ (Fig. 1a). Samples were digitised mainly with an imaging resolution of ~0.25 mm/px (*n* = 497), except for 18 samples scanned at ~0.50 mm/px, which were excluded (Fig. 1b and Supplementary Table S1). A feature matrix of all available data was built, consisting of only numeric or binary features as rows, with missing data reported as NA. Categorical variables were transformed into binary factors.

The somatic mutation calls (SNVs and indels) were collected from the Supplementary Table S1 by Ricketts et al. [22]. Briefly, the dataset was assembled using six different algorithms (MuTect, MuSE, Pindel, Somatic Sniper, VarScan2, and Radia) from four centres. Mutation calls were not available for all patients with transcriptome and clinical data.

The Helsinki dataset is composed of 64 ccRCC patients (Fig. 1a). For these, diagnostic H&E-stained histopathological slides have been digitised ~0.25 mm/px. Moreover, a tissue microarray has been previously prepared and stained with 6-plex T cell antibody panels using multiplex immunohistochemistry [13].

### Image annotation strategy

TCGA H&E slides have been stained at distinct participating clinical sites and scanned with varying resolution and digital scanner types into SVS format. Helsinki H&E slides have been acquired in MIRAX format. To ensure algorithm generalisability, we included annotations from all clinical centres.

First, we determined the main texture classes to annotate. We prioritised classification reliability and therefore minimised the number of tissue classes as often tiles included multiple texture types and some histological patterns commonly co-occurred, for example smooth muscle, fibrous stroma, and blood vessels. In addition, some patterns were challenging to differentiate reliably from individual tiles without larger context information for example torn, adipose, and necrotic tissue. We ended up with the following texture classes: renal cancer (“cancer”; *n* = 13,057 image tiles, 24.8% of all tiles); normal renal (“normal”; *n* = 8652, 16.4%); stromal (“stroma”; *n* = 5460, 10.4%) including smooth muscle, fibrous stroma and blood vessels; red blood cells (“blood”; *n* = 996, 1.9%); empty background (“empty”; *n* = 16,026, 30.4%); and other textures including necrotic, torn, and adipose tissue (“other”; *n* = 8522, 16.2%; Fig. 1c). We annotated in total 52,713 randomly selected tiles sized 300 × 300 px located in the centre of a larger 900 × 900 px image to improve the annotation accuracy. Images from both TCGA and Helsinki datasets were used in both training and test datasets to ensure that models are robust to sampling, digitisation and image format variability.

To quantify lymphocytes, we annotated 256 × 256 px tiles (*n* = 25,095) containing none or few lymphocytes as “Low” (*n* = 20,092, 80.1%) and the rest as “High” (*n* = 5,003, 19.9%; Fig. 1d). As areas of high lymphocyte density were substantially less common, we speeded up annotation by extracting regions of high lymphocyte aggregates and from regions of low lymphocyte infiltrate using the open-source software QuPath [23] 0.2.0. We selected ~20 digital TCGA samples originating from various clinical sites. All texture and lymphocyte images were evaluated twice to minimise annotation errors.

### Texture classification

For texture classification, we trained a multi-class CNN. We employed the deep residual network ResNet as it has been commonly used in computer vision tasks [24]. Transfer learning is the process of repurposing parameters of a previous algorithm to optimise training on a new dataset [25]. Here, we adapted transfer learning by combining the ImageNet-pretrained ResNet-18 infrastructure with a fully connected layer, a rectified linear activation function (ReLU) activation and a softmax layer for prediction. Training occurred at all CNN layers, with the Adam optimiser tuned with a fixed learning rate of 10^−4^, batch size 4, and the cross-entropy loss function until the validation loss did not decrease for 5 consecutive epochs. We randomly cropped 256 × 256 px tiles from the annotation images and augmented these with horizontal–vertical rotation and without balancing texture classes. Models were composed with Python 3.9.1. with libraries Pytorch 1.9, Torch 1.11.0, and Torchvision 0.12.0.

The classification resulted in tessellated texture areas (Supplementary Fig. S1). For instance, cancer regions were disrupted by sporadic tiles of other textures. To smooth texture masks, we slid the 3 × 3 tiles’ window size and stride of 2 over the texture map and unified the texture class in each window by the most common texture. In some occasions, two tissue textures occurred equally often for instance 4 cancer, 4 stroma and 1 blood tiles. If the most common class was a tie between cancer and another texture, the cancer class was prioritised. If the most common class was a tie between stroma and another texture, the stroma class was prioritised except if the other was cancer. Stroma and cancer textures were prioritised as these occurred most in tiles of multiple textures. In other cases of tie, the pooled texture type was randomly selected from the equally most occurring textures.

### Lymphocyte classification

For the lymphocyte classification, we trained a binary-class CNN using the same model infrastructure and hyperparameters as was used in the texture classification. However, to quantify the lymphocyte infiltration in a continuous range [0–1], we used the argmax function on the sigmoid layer. Therefore, no post-pooling of lymphocyte masks was performed.

### Tumour margin

To analyse the histological and lymphocyte content immediately exterior to the tumour, we defined the tumour margin as the first two non-cancer tiles around each cancer tile with the maximum_filter function of the Python Scipy 1.8.1. library. The margin was 512 px or ~128 μm wide. For reference, the average lymphocyte diameter is ~10 μm. The remaining tissue not included as tumour or tumour margin was classified as the non-margin tissue. To avoid sampling bias, we included only samples with ≥1% normal texture.

### Model metrics

We divided annotation datasets into training (70%), validation (20%), and test (10%) sets. The final model fitness was evaluated in the test set by comparing classification accuracy and a confusion matrix.

### Statistical analysis

Median and interquartile ranges (IQRs) were used to report average values and ranges. We compared two continuous variables with the Wilcoxon rank-sum test (unpaired, two-tailed) and three or more continuous variables with the Kruskal–Wallis test. We compared categorical variables with the *χ*^2^ test. To adjust *p* values, we used Benjamini–Hochberg correction. We evaluated 5-year overall survival with the Kaplan–Meier analysis (log-rank test). We limited comparisons with transcriptomic data to the protein-coding genes with a median expression value of >8 CPM (*n* = 9645). For pathway analyses, we included the Chromosome, Hallmark, PID, Reactome, Biocarta, and KEGG gene sets v.6.2. We performed statistical analyses with R 3.5.1.

### Textural mapping of lymphocyte infiltration

The texture-specific lymphocyte infiltration varied by clinical centres partly due to differing texture proportions reflecting sampling conventions. We harmonised total lymphocyte infiltration in two steps. First, we multiplied the proportion of each texture area by its relative lymphocyte proportion. The relative lymphocyte proportion reflects the lymphocyte enrichment to each texture in comparison to other textures. Thus, the sum of relative lymphocyte proportion in blood + cancer, + normal + blood + other = 100%. In the second step, we divided the sum of the normalised lymphocyte proportions by the total sample area, e.g.

$$\alpha = \frac{{\mathop {\sum }\nolimits_t^T \rho _tA_t}}{{\mathop {\sum }\nolimits_t^T A_t}}$$

where *ρ* represents the relative lymphocyte proportion (%) and *A* the area (px) of the texture *t*, which is part of the texture list *T* ∈ [blood, cancer, normal, stroma, other].

To fully normalise staining differences, we categorised samples into “High” and “Low” infiltration based on their lymphocyte density compared to the clinical centre median density. Small batch size increases the high risk for nonparametric data distribution. Therefore, we included only centres with more than 20 samples to the lymphocyte analyses.

## Results

### CNNs can reliably detect tissue textures and lymphocyte proportion

We trained CNNs to detect textures and lymphocyte infiltration in H&E-stained diagnostic tissue sections of ccRCC patients in two datasets (Fig. 1a, b). First, we thoroughly annotated 52,713 image tiles to one of six distinct texture classes and 25,095 tiles to low or high lymphocyte classes (Fig. 1a–d). The 18-layered ResNet (ResNet-18) correctly classified 95.0% of lymphocyte tiles in the test set (*n* = 2510). The ResNet-34 achieved 95.7% and the ResNet-50 95.3% classification accuracy indicating that increasing model depth slowed training but did not improve model fitness. Detailed classification metrics are visualised in Fig. 1e and Supplementary Fig. S2.

For consistency, we used the ResNet-18 infrastructure also for computational texture mapping (CTM). The algorithm achieved 94.5% total classification accuracy in the test set (*n* = 5272) ranging from 75.2% for blood texture to 98.2% to empty areas (Fig. 1e). Most of the classification errors were related to tiles composed of a mixture of textures. For instance, haemorrhage is a typical histopathological finding in ccRCC due to neovascularisation and structural instability of rapidly growing blood vessels [26]. Therefore, the misclassification of blood tiles as Other texture was observed due to their co-occurrence with torn tissue. The algorithm misclassified only 10 out of 1335 (0.7%) cancer texture images as normal renal tissue and 8 out of 822 (1.0%) normal renal tissue images as renal cancer indicating excellent distinction.

### TCGA clinical sites differ by their texture characteristics

TCGA ccRCC samples have been collected from 16 participating clinical sites. The tissue procurement protocol has been previously described [14]. However, the actual sampling uniformity remains unknown, although this may significantly hamper the integrity of TCGA studies.

We used first CTM results to compare texture composition of samples by their clinical site. We observed 2.3-fold variation (36.4-84.0%) in the median proportion of cancer texture (Fig. 1f, g). Consistently, we noted substantial differences also in other texture types (Supplementary Fig. S3). Samples with >5% median normal renal proportion originated from MD_Anderson_Cancer_Center and MSKCC indicating centre-specific conventions to collect intratumoural or a mixture of intra-peritumoural samples (Fig. 1f and Supplementary Fig. S3).

To further measure data quality, we examined in detail samples with <5% cancer texture and excluded 51 samples from 48 TCGA patients (9.6%; Fig. 1b and Supplementary Table S1). CTM provided excellent functionality for quality control as the reason for low cancer proportion was atypical or non-ccRCC histology (*n* = 22), poor histological quality (*n* = 21), necrotic sample (*n* = 4), and lack of cancer tissue (*n* = 4). In summary, CTM can be used to identify sampling bias, technically suboptimal images and non-ccRCC samples prior to other histopathological analyses.

### Tissue haemorrhage is associated with lower metastasis rate, less frequently mutated mTOR, and lower infiltration of regulatory T cells

**Fig. 2:**
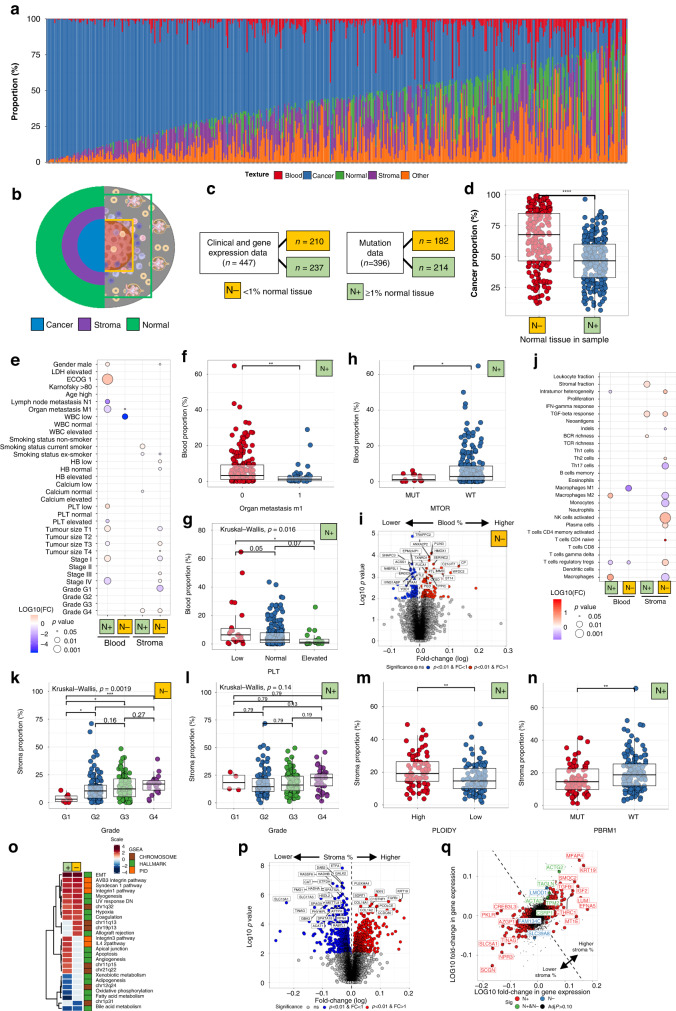
Tissue texture analysis. **a** Tissue texture profiles in individual patients (*n* = 447). **b** Schematic of the clear-cell renal cell carcinoma microenvironment. The left side illustrates three common subregions: the intratumoural cancer tissue, the stroma-rich margin and the outer normal renal tissue. The right side illustrates two examples of sampling conventions: without (green, “N−”) or with (light blue, “N+”) normal renal tissue. **c** Patient numbers by normal tissue proportion. **d** Cancer textures by N+ or N− samples. **e** Association between clinical variables (binary variables) and textures (continuous variables). **f** Blood texture by organ metastasis, **g** peripheral blood platelet and **h** *mTOR* mutation in N+ samples. **i** Genes differentially expressed in tumours with higher (right) or lower (left) than median blood proportion. **j** Association between transcriptome-based signatures (binary variables) and textures (continuous variables). **k** Stroma by tumour Fuhrman grade in N− and **l** N+ samples. **m** Stroma proportion by ploidy status in N+ samples. **n** Stroma proportion by *PBRM1* mutation status in N+ samples. **o** Heatmap of the normalised enrichment score of the significant gene pathways associated (adjusted *p* < 0.05) with N+ and N− samples. Grey-coloured boxes indicate the pathways associated with only N+ or N− samples (adjusted *p* < 0.05). **p** Genes differentially expressed in tumours with higher (right) to lower (left) than median stroma proportion. Only N+ samples are included. **q** Preferential association with higher stroma tissue proportion in N+ or N− samples.

Next, we aimed to resolve the textural landscape of ccRCC patients and its clinical and molecular correlates. As expected, renal cancer tissue was the most prevalent texture (median 54.3%), followed by stroma (14.3%), other (9.6%), blood (2.3%) and normal tissue (1.3%; Fig. 2a and Supplementary Fig. S4A). In total, in 191/447 (42.7%) samples the tissue section was covered by less than 50% of cancer cells implying high textural heterogeneity. In the Helsinki dataset, the distribution was nearly identical (Supplementary Fig. 4A, B). Normal renal tissue was the second most frequent texture as the dataset was designed to cover tumour border. The proportion of samples with >50% cancer tissue was 48.4%, which is in line with TCGA dataset.

Besides tumour biology, the varying site-specific sampling protocols likely affected the textural content. We reasoned that by dividing TCGA ccRCC samples by absence (<1%, “N−”) and occurrence of normal tissue (≥1%, “N+”) we could undermine the variation and examine tissue textures in two histologically more coherent cohorts (Fig. 2b, c). N− samples were composed of more cancer texture (median 67.7% [IQR 46.5-84.9%]) representing the tumour core compared with (46.6% [33.0-60.0%]) in N+ samples reflecting a broader tumour microenvironment (Fig. 2d and Supplementary Fig. S4C–E).

As the proportion of normal renal tissue reflected tissue sampling practices and the “other” texture class included various histological types, we focused on blood and stroma-associated phenotypes. Higher haemorrhage in N+ samples was associated with less frequent metastasis, lower tumour stage and superior Eastern Cooperative Oncology Group (ECOG) performance status (Fig. 2e, f). Yet, no association with survival was evident (Supplementary Fig. S5). We also noted that the peripheral blood (PB) platelet count gradually decreased with increasing proportion of tumour haemorrhage in N+ samples (Fig. 2g). Elevated pretreatment platelet level is a biomarker of poor survival and is incorporated in the prognostic Heng score [27]. Thus, low PB platelet count could be due to high angiogenic activity and consequent tumour haemorrhage. When examining N− samples, we observed similar but less significant association of tumour haemorrhage suggesting peritumoural haemorrhage to have a more significant role in disease pathology (Fig. 2e).

We investigated next genomic alterations. In N+ samples, *mTOR*^mut^ occurred in 24/396 patients (6.1%) and associated with lower haemorrhage (Fig. 2h). While mTOR has been described to increase angiogenesis via HIF1α and VEGF-regulated pathways, *VHL*^mut^ was not associated with haemorrhage indicating another mechanism. No additional association with gene alterations, mutation burden or aneuploidy was observed in either N+ or N- samples (Supplementary Tables S2 and S3).

To identify haemorrhage-associated transcriptomic signatures, we first compared the expression of individual genes (Fig. 2i and Supplementary Fig. S6A). In N− samples, the *HMOX1* gene was highly expressed in conjunction with haemorrhage (Fig. 2i). Heme oxygenase 1 (*HMOX1)* catalysers heme to biliverdin and increased heme catabolism is consistent with increased tissue haemorrhage [28]. We observed upregulated epithelial-to-mesenchymal (EMT) and hypoxia-related pathways in samples without normal tissue but little difference in immune profiles (Fig. 2j and Supplementary Fig. S6B, C). In summary, our findings indicate that peritumoural and intratumoural haemorrhage differ by their clinical, mutational, transcriptional, and immunological profiles.

### Tissue fibrosis is associated with high histological grade, low mutation burden and an adaptive immune response

Next, we examined fibrosis-related manifestations. In N− samples, we observed association between stroma and histological grade (Fig. 2e, k). Similar relation was less evident in N+ samples indicating that intratumoural but not peritumoural stroma would be linked with poor renal cell differentiation (Fig. 2l). In line, N- stroma associated with other established adverse prognostic biomarkers such as tumour size, stage and anaemia (Fig. 2e).

When studying genomic alterations in N+ samples, *PBRM1*^wt^ and diploid haplotype were associated with higher proportion of stroma (Fig. 2m, n and Supplementary Table S4). In N− samples, *SETD2*^mut^ indicated lower fibrosis (Supplementary Table S5).

Fibrosis in N− samples was associated with fewer macrophages and enrichment of regulatory T cells and activated NK-cells (Fig. 2j and Supplementary Fig. S6D–F). As mutation burden and *PBRM1* genotype have been implicated with immunotherapy response, quantifying fibrosis could provide an inexpensive biomarker to increase their precision or select patients for targeted sequencing [29–31].

Next, we inspected fibrosis-associated transcriptional programmes. As expected, the TGFβ response pathway was enriched both in N+ and N− samples with high stromal composition (Fig. 2j). High histological stroma associated only in N+ samples with transcriptome-derived stromal score (Fig. 2j). We reasoned this to be due to more abundant stroma in N+ compared to N− samples as visually confirmed by large peritumoural stromal margins compared to intratumoural fibrotic islets (Supplementary Fig. S7A).

Established stromal signalling pathways regulating EMT and the formation of integrin, syndecan, and myogenesis were elevated in both N+ and N− samples (Fig. 2o). Moreover, fibrosis was associated with chromosomal locus 1q32 activation, coagulation and hypoxia (Fig. 2o). The apoptosis pathway was overactivated and active lipid metabolism decreased only in conjunction with enriched stroma in N+ samples (Fig. 2o). Similar findings were observed also at the gene-level (Fig. 2p and Supplementary Fig. S7B). While some genes were associated with stroma formation in both sample categories, transforming growth factor, beta-induced (*TGFBI*) and cytokeratin 19 (*KRT19*) were enriched in N+ samples whereas actin gamma 2, smooth muscle (*ACTG2*) in N− samples. These findings suggest that the extracellular matrix (ECM) in the intratumoural and peritumoural tissue could differ by their adhesion, migration and cell signalling abilities (Fig. 2q).

### Lymphocyte infiltration is coordinated between malignant and surrounding tissue

Previous studies quantifying tumour lymphocyte infiltration in ccRCC have relied on deconvolution of bulk RNA-sequencing data [17], histochemical [32], or antibody-based detection such as flow cytometry [33, 34]. While these approaches are precise to approximate the lymphocyte population in a sample, they impose demands on uniform sampling and sample processing.

**Fig. 3:**
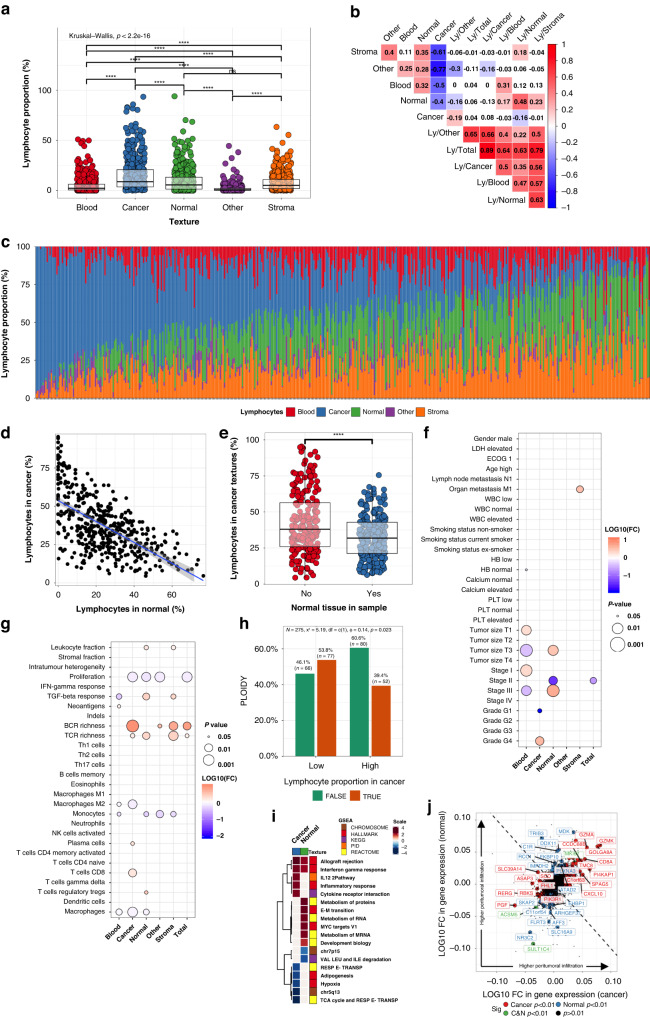
Texture-specific lymphocyte analysis. **a** Lymphocyte proportion by textures. **b** Correlation matrix of the texture and texture-specific lymphocyte proportion. **c** Relative lymphocyte proportion (density normalised to 100%) by textures in individual patients (*n* = 447). The lymphocyte density (proportion/area) has been median-averaged by texture type and then their sum rescaled to 100%. **d** Correlation of relative lymphocyte proportion in malignant and normal renal tissue. **e** Lymphocyte proportion in cancer tissue by samples with or without normal tissue. **f** Association between clinical variables (binary variables) and texture-aware lymphocyte proportions (continuous variables). **g** Association between transcriptome-based signatures (binary variables) and texture-specific lymphocyte proportions (continuous variables). **h** Ploidy status (FALSE = aneuploidy, TRUE = normal) by lymphocyte infiltration in cancer tissue. **i** Heatmap of the normalised enrichment score of the significant gene pathways associated (adjusted *p* < 0.05) with lymphocyte infiltration to cancer or normal textures. Grey-coloured boxes indicate the pathways associated with only samples with or without normal tissue (adjusted *p* < 0.05). **j** Genes associated with lymphocyte infiltration to the cancer (red) or normal (blue) or both (green) texture. The coordinate of each dot represents the ratio of the gene expression value when comparing in (cancer or normal) texture-specific lymphocyte-rich vs. lymphocyte-low samples.

Here, we built a deep learning model identifying images with high (90.3% classification accuracy) and low (96.1%) lymphocyte density in the test set (Fig. 1e). We hypothesised that CTM-guided lymphocyte quantification could solve issues related to sampling variation. The lymphocyte classification probability per tile reflected lymphocyte density. Therefore, lymphocyte predictions [0–1] were proportioned by texture surface area. The median lymphocyte proportion per sample was 20.3% and varied between 2.3–82.5%. The highest lymphocyte density was unexpectedly in the normal renal texture followed by cancer, stroma, blood, and lastly other texture types (Fig. 3a). While texture-specific lymphocyte proportions shared high positive correlation, intratumoural infiltration explained most of the total sample infiltration variance (Fig. 3b).

To examine lymphocyte textural priority, we rescaled the texture-specific lymphocyte densities so that their sum would equal to 100% and observed heterogeneous distribution at the patient level (Fig. 3c). Strikingly, the intratumoural lymphocyte density correlated negatively with the density in the surrounding normal renal tissue (*R* −0.58, *p* < 0.001; Fig. 3d) and stromal texture (*R* −0.51, *p* < 0.001). Collectively, the results indicate that lymphocyte infiltration affects all textures but unequally possibly reflecting flow intratumoural and peritumoural areas.

To confirm the generalisability of the lymphocyte classifier and the stability of the lymphocyte proportion per textures, we were able to replicate all results depicted in Fig. 3a–d in the Helsinki dataset (Supplementary Fig. S8).

### Stromal lymphocytes are associated with poor survival and high T cell receptor diversity

Lymphocyte proportions in the cancer and stroma textures differed by the proportion of normal renal tissue (<1% vs. ≥1%; Fig. 3e and Supplementary Fig. S9A–C). To equalise sampling differences, we normalised lymphocyte density with CTM-derived texture-specific weights (see Methods) successfully reducing differences in the total lymphocyte infiltration by clinical centre (Supplementary Fig. S9D, E). However, samples originating from Fox Chase contained higher lymphocyte density than expected (Supplementary Fig. S9E). When examined visually, these samples were characterised with a high hematoxylin:eosin ratio and lymphocyte scoring even visually was ambiguous (Supplementary Fig. S9F, G). As a conclusion, these samples were excluded from lymphocyte analyses.

To fully normalise staining differences, we categorised samples into “High” and “Low” infiltration based on their lymphocyte density compared to the clinical centre median density. When examining clinical significance, we observed that lymphocyte infiltration in cancer textures was associated with high histological grade (Fig. 3f). Stromal lymphocyte infiltration was related with poor overall survival, and organ metastasis (Fig. 3f and Supplementary Fig. S10). Instead, high blood-specific lymphocyte density and low normal renal-specific lymphocyte density were associated with more local tumour but not survival (Fig. 3f and Supplementary Fig. S10). Patient gender, age, smoking status, or laboratory values were not related to lymphocyte infiltration (Fig. 3f).

We then studied genomic and immunological profiles. In line with our expectations, infiltration in malignant renal tissue was associated with the transcriptomic CD8+ T cell signature (Fig. 3g). B and T cell receptor diversity was associated with increased lymphocyte infiltration almost irrespective of textural context (Fig. 3g). Of note, cell proliferation correlated negatively with all except stromal and blood lymphocyte infiltration (Fig. 3g).

### Aneuploidy, chromosome 1p and 5q loci, and the EMT programme identified as regulators of tumour-infiltrating lymphocytes

Next, we examined genomic alterations associated with lymphocyte infiltration. Aneuploidy but not mutation burden was the most reliable predictor of high intratumoural and total lymphocyte density (Fig. 3h and Supplementary Fig. S11A). When studying individual genes, *SETD2*^mut^ was associated with higher infiltration in stromal texture (Supplementary Fig. S11B). The *PBRM1* genotype has been associated with both nonimmunogenic [31] and immune hot phenotype and anti-PD1 therapy response [30]. In our study, *PBRM1* alterations was associated only with lymphocyte infiltration in normal renal tissue (Supplementary Fig. S11C–E). By analysing the supplementary data provided in [30], no association was evident between the tumour core CD8+ density and *PBRM1* status validating our finding and indicating that its immunologic significance remains unclear (Supplementary Fig. S11F).

Given our previously described asynchronous lymphocyte enrichment in either malignant or normal renal tissue, we interrogated which pathways were most commonly altered in these two compartments. The top three pathways enriched with intratumoural infiltration were well-established T cell activation signatures endowing confidence to our analysis (Fig. 3i). At the gene-level, the cytolytic granzyme A (*GZMA*) and K (*GZMK*) enzyme, *CD8A* and the chemokine *CXCL10* illustrious of T and NK-cells and the testis antigen *SPAG5* were associated with infiltration to cancer tissue (Fig. 3j). Lipid metabolism, hypoxia and chromosome locus 5q13 genes were downregulated in samples with high intratumoural infiltration. On the contrary, the hallmark EMT and protein metabolism pathways were enriched in lymphocyte-rich normal renal tissue (Fig. 3i). In summary, immune, mesenchymal, and metabolic factors influence lymphocyte infiltration.

### The textural composition of the tumour margin predicts prognosis and reflects the tumoural genomic and transcriptomic alterations

**Fig. 4:**
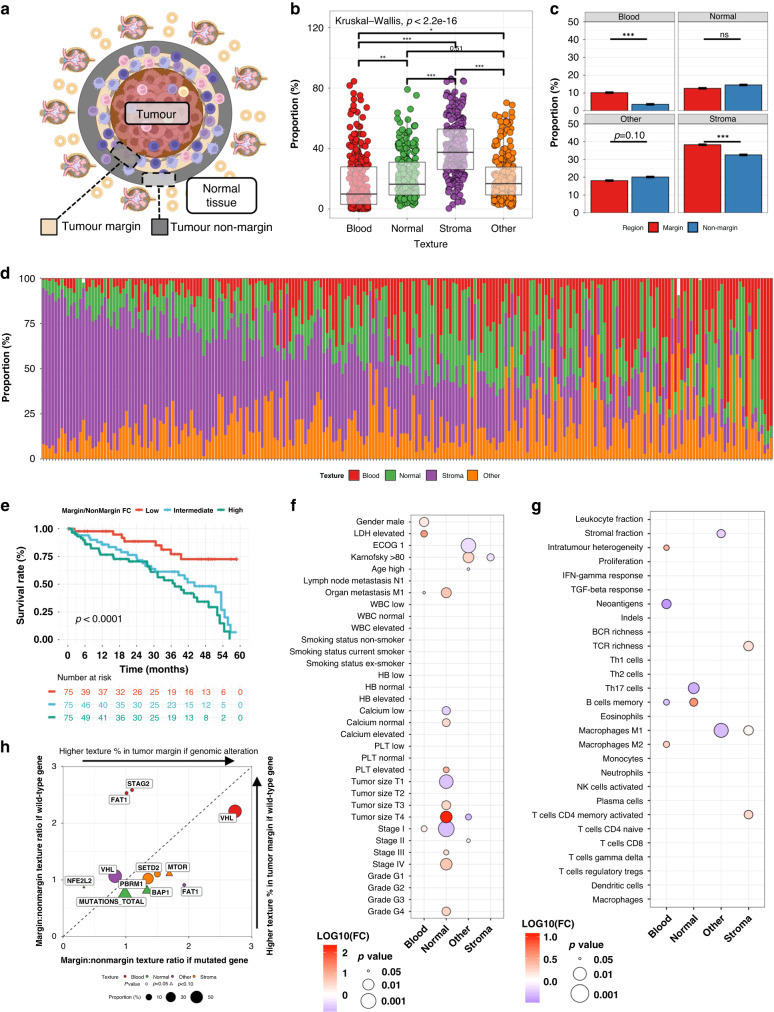
Texture profile in the tumour margin. **a** Schematic of the peritumoural tissue. **b** Tissue texture proportions in the tumour margin, and **c** comparison to the non-margin tissue. **d** Relative texture proportion in the tumour margin in individual patients (*n* = 447). **e** Kaplan–Meier curves by the margin:non-margin ratio of normal tissue proportion. **f** Association between clinical variables (binary variables) and the margin:non-margin texture ratios (continuous variables). **g** Association between transcriptome-based signatures (binary variables) and the margin:non-margin texture ratios (continuous variables). **h** Enrichment of genomic alterations by margin:non-margin texture ratios. Only associations with *p* < 0.10 are visualised.

Based on our previous findings that the tumour core and its surrounding peritumoural tissue form two immunologically distinct regions [13]. Therefore, we were intrigued to investigate the textural content of the peritumoural margin and its exterior non-margin region (Fig. 4a). The peritumoural texture was dominated by stroma (Fig. 4b). Blood and stroma textures were more frequent in the tumour margin and normal renal tissue less common in the tumour margin than in its exterior non-margin (Fig. 4c).

The margin composition was heterogenous at the patient level (Fig. 4d). We assigned each patient with a textural enrichment score by comparing texture proportions between the tumour margin to the non-margin tissue. Patients with a high margin:non-margin normal renal tissue ratio had significantly worse survival (Fig. 4e). The clinical profile of these patients included less differentiated tumour and advanced stage (Fig. 4f). These tumours were enriched for memory B cells and depleted of Th17 helper T cells (Fig. 4g). In addition, these were characterised with more frequent mutations in *PBRM1*, *SETD2* and *mTOR* genes and activation of the *KRAS* pathway and inflammatory signalling (Fig. 4h and Supplementary Fig. S12A, B).

In the opposite, patients with elevated blood texture in the tumour margin were characterised with superior survival (Supplementary Fig. S12C). These were commonly male patients with local tumours and mutations in *VHL* and wild-type *FAT1* and *STAG2* (Fig. 4f, h). Based on transcriptomic data, these tumours were associated with activation of the antigen processing pathways and fewer neoantigens (Fig. 4g and Supplementary Fig. S12D, E).

High peritumoural margin:non-margin stroma was associated with T cell clonal diversity and mutations in *PBRM1*, *SETD2* and *mTOR*, but no distinct prognostic or transcriptomic signature (Fig. 4f–h and Supplementary Fig. S12F–H).

### The lymphocyte-rich stromal margin associates with an adaptive immune response, dampened EMT and non-smoking habit

**Fig. 5:**
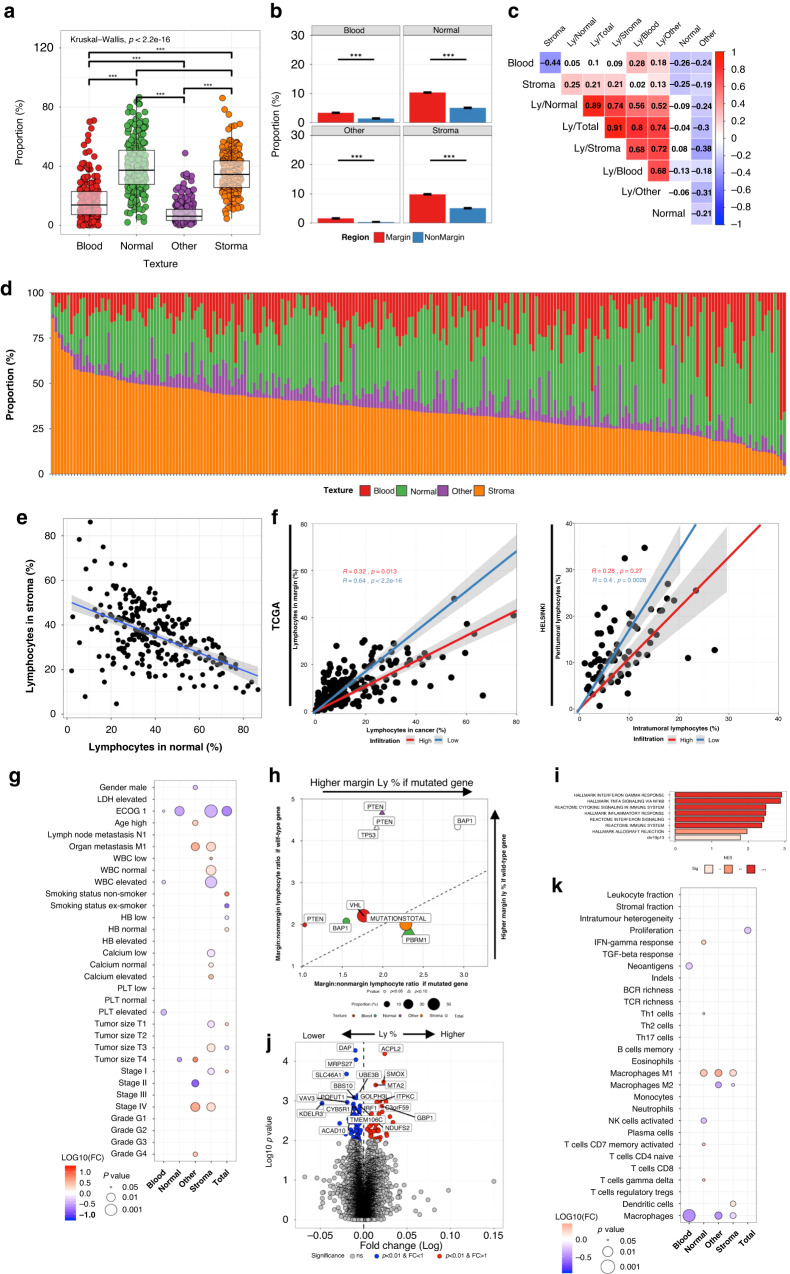
Lymphocyte infiltration in the tumour margin. **a** Lymphocyte proportion in the tumour margin by textures, and **b** comparison to the non-margin tissue. **c** Correlation matrix of the texture and texture-specific lymphocyte proportion in the tumour margin. **d** Relative lymphocyte proportion (density normalised to 100%) by textures in the tumour margin of individual patients. The lymphocyte density (proportion/area) has been median-averaged by texture type and then their sum rescaled to 100%. **e** Correlation of relative lymphocyte proportion in stromal margin and normal renal margin. **f** Correlation between the lymphocyte density in the cancer texture and tumour margin (TCGA dataset) and in the intratumoural and peritumoural regions (Helsinki dataset). Patients are divided by highest 25% (red) and lowest 75% (blue) cancer texture-specific lymphocyte density. **g** Association between clinical variables (binary variables) and the margin:non-margin ratio of lymphocyte proportion (continuous variables). **h** Enrichment of genomic alterations by margin:non-margin ratio of lymphocyte proportions in tissue textures. Only associations with *p* < 0.10 are visualised. **i** Normalised enrichment score (NES) of the gene pathways significantly associated with the ratio of margin:non-margin stroma proportion. **j** Comparison of the genes differentially-expressed in tumours with higher (right) or lower (left) margin lymphocyte density compared to non-margin lymphocyte density. **k** Association between transcriptome-based signatures (binary variables) and the margin:non-margin ratio of lymphocyte proportion (continuous variables).

To conclude, we quantified the margin:non-margin lymphocyte ratio. The highest lymphocyte density was found in the normal renal tissue and stroma in both margin and non-margin tissues (Fig. 5a). However, lymphocytes were more abundant in the tumour margin across textures when compared to the non-margin tissue indicating their enrichment to the close proximity of tumours (Fig. 5b). While absolute lymphocyte infiltration correlated regardless of the histological texture they were located in, we noted distinct negative correlation between infiltration to normal renal and stromal tissues (Fig. 5c–e).

Next, we correlated intratumoural and peritumoural lymphocyte density and found strong concordance (*R* = 0.78, *p* < 0.001, Fig. 5f). We replicated the finding in the Helsinki dataset, where instead of a cancer texture and its margin we had annotated the intratumoural and peritumoural regions (*R* = 0.60, *p* < 0.001, Fig. 5f). However, in closer inspection we could discern two linearities and, therefore, divided patients into two groups: 25% highest and 75% lowest tumoural lymphocyte density. Patients with high intratumoural infiltration had lower peritumoural lymphocyte density. Collectively, the findings suggest that distinct mechanisms could regulate immune cell activation (infiltration to all textures), their spatial localisation to distinct areas of the tumour border, and penetration from the border to the tumour core.

Then, we studied interrogated the clinical and genomic profiles of the tumour margin infiltration. Enrichment of lymphocytes to the tumour border was associated with inferior survival (Supplementary Fig. 13). The finding was related especially to infiltration to the margin characterised with stromal and other textures which were associated with biomarkers of poor prognosis (e.g., tumour size, stage, metastasis, poor ECOG; Fig. 5g). We also identified wild-type *BAP1* to associate with higher margin:non-margin lymphocyte ratio in both normal renal tissue and in general, while enrichment of lymphocytes to the stromal margin was associated with higher mutation burden (Fig. 5h).

We observed pronounced interferon and adaptive immune signalling signatures and higher M1-polarisation of macrophages to correlate with lymphocyte enrichment to the tumour margin (Fig. 5i–k and Supplementary Fig. 14). To further explore immune profiles, we studied multiplex immunohistochemistry-stained tissue samples of the Helsinki dataset, which has been constructed of renal sections encompassing the tumour border. We compared the T cell immunophenotypes of patients with high (*n* = 31) vs. low (*n* = 31) margin:non-margin lymphocyte ratio. Intratumoural T cells did not differ immunophenotypically between these groups (Supplementary Fig. 15). Instead, margin lymphocyte enrichment was associated with lower granzyme B expression in peritumoural CD4 + T cells but higher CD25 expression in CD8 + T cells suggesting possible cytotoxic T cell activation (Supplementary Fig. 15).

## Discussion

The main findings of this study are (1) CTM to resolve sampling variation and (2) the integrative network between tissue textures, lymphocyte infiltration, clinical variables, genomic alterations, and transcriptomic signatures.

TCGA tissue procurement has been described [14], but limitedly evaluated [35]. Here, we indicate that some centres have systematically included samples spanning from the tumour core to the surrounding healthy tissue while others have restricted sampling to the tumour core. Moreover, the hematoxylin:eosin ratio differed substantially by clinical centres as previously reported [35]. While sampling differences are apparent in TCGA histological slides, these could extend to sequencing data urging for uniform sampling, sample preprocessing, and retrospective evaluation standards.

While we could undermine staining variance by configuring algorithm training, we resolved sampling variation with CTM (Fig. 4). The approach detected up to 10% samples representing false histology or poor quality. In addition, CTM abrogated lymphocyte infiltration differences in distinct clinical centres. The approach could be beneficial as a standardisation step in diverse applications of computational histopathology such as dataset quality control and harmonisation.

**Fig. 6:**
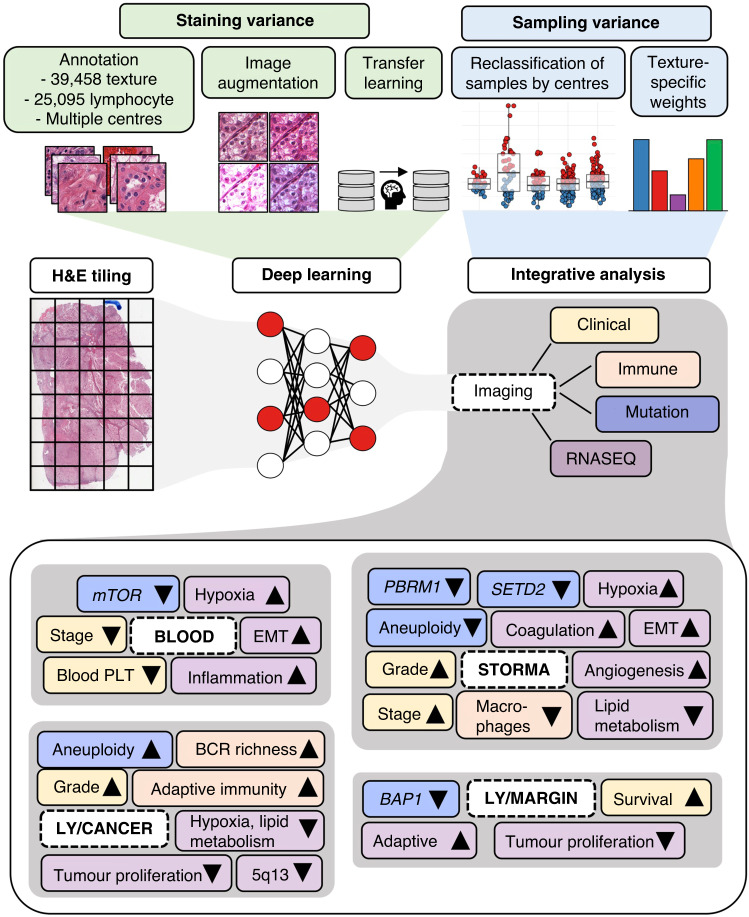
Summary of the study. H&E haematoxylin and eosin, EMT epithelial-to-mesenchymal transition, PLT platelet, BCR B cell receptor, TCR T cell receptor, Ly lymphocyte density.

We demonstrate the generalisation of CTM in two datasets and its usability by uncovering multifaceted connections between texture and lymphocyte density with clinical, immunological, genomic, and transcriptomic features. For instance, while the histological grade is based on ccRCC cell morphology [36, 37], it correlated substantially with intratumoural fibrosis, which could facilitate routine tumour grading. Fibrosis correlated with immune, hypoxia and EMT signalling and alterations in commonly mutated genes (Fig. 6). Half of the lymphocytes in sections were located among cancer cells and shared moderate concordance across textures. Aneuploidy, the IL-12 pathway, and chromosome 5q13 inactivity were discovered as novel biomarkers of intratumoural infiltration in ccRCC.

We have previously shown that intratumoural T cells are immunophenotypically more experienced based on higher expression of cytolytic, immune checkpoint and senescence markers and closer intercellular proximity compared to the peritumoural and normal renal regions [13]. Possibly due to restricted penetration, lymphocyte enrichment to the stromal margin was associated with advanced tumour stage (Fig. 6). In addition, we discovered that while normal renal tissue is commonly found in the tumour margin, it is implicated with poor survival emphasising the role of a buffer between malignant and normal renal tissue.

The comprehensive annotation data and algorithms are available to expand the textural and lymphocyte analyses to other datasets. In summary, this study highlights how computational analysis of routine H&E staining can help to detect sampling fallacies and poor-quality samples easily to discover novel associations between histopathology and molecular correlates.

## Supplementary information

Supplemental tables

Supplemental figures

## Data Availability

The annotated texture and lymphocyte image data and algorithm parameters have been deposited to Zenodo https://zenodo.org/record/7898308#.ZGXM3-xBxAc. The TissUUmaps [38] visualisation platform is available at http://hruh-20.it.helsinki.fi/rcc_texture_lymphocytes/.

## Code availability

The code to run the CNN classifiers are available at https://github.com/vahvero/RCC_textures_and_lymphocytes_publication_image_analysis. The code and processed data to reproduce data analyses and figures are available at https://github.com/obruck/RCC_textures_and_lymphocytes_publication_data_analysis.
