# Supplementary material for: Computational textural mapping harmonises sampling variation and reveals multidimensional histopathological fingerprints
Source: Br J Cancer. 2023 Jun 30;129(4):683–95. doi: 10.1038/s41416-023-02329-4 (PMC10421901; doi:10.1038/s41416-023-02329-4)
Supplement: Supplementary file 1 — Supplemental tables [file 41416_2023_2329_MOESM1_ESM.docx]

Supplementary data for Brummer O. *et al*, Computational Textural Mapping Harmonizes Sampling Variation and Reveals Multidimensional Histopathological Fingerprints

Supplementary table 1. Samples excluded from analyses.

| **TCGAid** | **errortype** |
| --- | --- |
| TCGA-B8-5158 | low resolution |
| TCGA-B8-5159 | low resolution |
| TCGA-B8-5163 | low resolution |
| TCGA-B8-5164 | low resolution |
| TCGA-B8-5165 | low resolution |
| TCGA-BP-4770 | low resolution |
| TCGA-CZ-4853 | low resolution |
| TCGA-CZ-4854 | low resolution |
| TCGA-CZ-4856 | low resolution |
| TCGA-CZ-4857 | low resolution |
| TCGA-CZ-4858 | low resolution |
| TCGA-CZ-4859 | low resolution |
| TCGA-CZ-4861 | low resolution |
| TCGA-CZ-4862 | low resolution |
| TCGA-CZ-4863 | low resolution |
| TCGA-CZ-4864 | low resolution |
| TCGA-CZ-4865 | low resolution |
| TCGA-CZ-4866 | low resolution |
| TCGA-AK-3456 | necrosis |
| TCGA-BP-4804 | necrosis |
| TCGA-BP-4167 | necrosis&poor quality |
| TCGA-CJ-5671 | necrosis&poor quality |
| TCGA-A3-A8OV | no cancer |
| TCGA-A3-A8OX | no cancer |
| TCGA-B8-4619 | no cancer |
| TCGA-GK-A6C7 | no cancer |
| TCGA-A3-3328 | not ccrcc |
| TCGA-AK-3427 | not ccrcc |
| TCGA-AK-3427 | not ccrcc |
| TCGA-AK-3433 | not ccrcc |
| TCGA-AK-3440 | not ccrcc |
| TCGA-AK-3443 | not ccrcc |
| TCGA-AK-3447 | not ccrcc |
| TCGA-AK-3453 | not ccrcc |
| TCGA-AK-3465 | not ccrcc |
| TCGA-B0-4699 | not ccrcc |
| TCGA-B0-4834 | not ccrcc |
| TCGA-B0-5083 | not ccrcc |
| TCGA-B0-5117 | not ccrcc |
| TCGA-B2-3923 | not ccrcc |
| TCGA-BP-4334 | not ccrcc |
| TCGA-BP-4994 | not ccrcc |
| TCGA-AS-3777 | not typical ccrcc |
| TCGA-B2-4101 | not typical ccrcc |
| TCGA-B8-A54E | not typical ccrcc |
| TCGA-T7-A92I | not typical ccrcc + weird stain |
| TCGA-T7-A92I | not typical ccrcc + weird stain |
| TCGA-T7-A92I | not typical ccrcc + weird stain |
| TCGA-B0-5120 | poor quality |
| TCGA-BP-5201 | poor quality |
| TCGA-CJ-4891 | poor quality |
| TCGA-CJ-4892 | poor quality |
| TCGA-CJ-4893 | poor quality |
| TCGA-CJ-4899 | poor quality |
| TCGA-CJ-5672 | poor quality |
| TCGA-CJ-5675 | poor quality |
| TCGA-CJ-5676 | poor quality |
| TCGA-CJ-5677 | poor quality |
| TCGA-CJ-5678 | poor quality |
| TCGA-CJ-5679 | poor quality |
| TCGA-CJ-5680 | poor quality |
| TCGA-CJ-5681 | poor quality |
| TCGA-CJ-5682 | poor quality |
| TCGA-CJ-5683 | poor quality |
| TCGA-CJ-5684 | poor quality |
| TCGA-CJ-5686 | poor quality |
| TCGA-CJ-5689 | poor quality |
| TCGA-CZ-5452 | poor quality |
| TCGA-BP-4782 | poor quality, low proportion |

Supplementary table 2. Association of genomic alterations and blood texture in samples with normal tissue.

| **genes** | **pvalue** | **Class 1 median** | **Class 2 median** | **Class 1 value** |
| --- | --- | --- | --- | --- |
| *MTOR* | 0.037 | 1.15 | 2.83 | Mutated |
| *KDM6A* | 0.15 | 9.19 | 2.68 | Mutated |
| *VHL* | 0.20 | 3.17 | 2.26 | Mutated |
| *SMARCB1* | 0.20 | 0.32 | 2.8 | Mutated |
| Mutations total | 0.22 | 2.47 | 3.18 | High burden |
| *BAP1* | 0.26 | 1.90 | 3.03 | Mutated |
| *FAT1* | 0.26 | 2.47 | 2.80 | Mutated |
| *PIK3CA* | 0.29 | 1.41 | 2.83 | Mutated |
| *KDM5C* | 0.33 | 4.85 | 2.77 | Mutated |
| *NF2* | 0.34 | 2.01 | 2.80 | Mutated |
| *SETD2* | 0.37 | 1.83 | 2.83 | Mutated |
| *PBRM1* | 0.52 | 2.63 | 2.79 | Mutated |
| *NFE2L2* | 0.67 | 2.77 | 2.80 | Mutated |
| Ploidy | 0.67 | 3.16 | 2.97 | Diploid |
| *TP53* | 0.75 | 2.84 | 2.77 | Mutated |
| *PTEN* | 0.80 | 2.65 | 2.79 | Mutated |
| *STAG2* | 0.81 | 3.06 | 2.77 | Mutated |

Supplementary table 3. Association of genomic alterations and blood texture in samples without normal tissue.

| **genes** | **pvalue** | **Class 1 median** | **Class 2 median** | **Class 1 value** |
| --- | --- | --- | --- | --- |
| Mutations total | 0.066 | 2.23 | 1.50 | Mutated |
| *KDM5C* | 0.098 | 3.85 | 1.80 | High burden |
| *PIK3CA* | 0.10 | 11.09 | 1.94 | Mutated |
| *NF2* | 0.16 | 0.08 | 1.97 | Mutated |
| *SMARCB1* | 0.21 | 0.15 | 1.97 | Mutated |
| *MTOR* | 0.36 | 1.51 | 1.96 | Mutated |
| *NFE2L2* | 0.41 | 4.16 | 1.94 | Mutated |
| *VHL* | 0.60 | 1.99 | 1.69 | Mutated |
| *PTEN* | 0.60 | 2.62 | 1.90 | Mutated |
| *PBRM1* | 0.63 | 1.98 | 1.94 | Mutated |
| *TP53* | 0.63 | 0.74 | 1.97 | Mutated |
| *SETD2* | 0.70 | 2.40 | 1.94 | Mutated |
| *FAT1* | 0.70 | 1.98 | 1.96 | Mutated |
| *KDM6A* | 0.74 | 1.23 | 1.97 | Mutated |
| *BAP1* | 0.76 | 1.55 | 1.99 | Mutated |
| *STAG2* | 0.91 | 3.27 | 1.96 | Mutated |
| Ploidy | 0.91 | 1.46 | 1.50 | Diploid |

Supplementary table 4. Association of genomic alterations and stroma texture in samples with normal tissue.

| **genes** | **pvalue** | **Class 1 median** | **Class 2 median** | **Class 1 value** |
| --- | --- | --- | --- | --- |
| Ploidy | 0.0019 | 19.16 | 14.63 | Mutated |
| *PBRM1* | 0.0083 | 14.59 | 18.73 | Diploid |
| Mutations total | 0.060 | 15.23 | 17.71 | High burden |
| *PTEN* | 0.090 | 23.75 | 16.38 | Mutated |
| *KDM6A* | 0.18 | 13.44 | 16.56 | Mutated |
| *STAG2* | 0.24 | 21.66 | 16.46 | Mutated |
| *NFE2L2* | 0.26 | 15.34 | 16.56 | Mutated |
| *VHL* | 0.30 | 16.06 | 17.37 | Mutated |
| *KDM5C* | 0.34 | 19.21 | 16.46 | Mutated |
| *BAP1* | 0.40 | 14.64 | 16.62 | Mutated |
| *MTOR* | 0.44 | 14.72 | 16.49 | Mutated |
| *SETD2* | 0.50 | 15.90 | 16.54 | Mutated |
| *PIK3CA* | 0.59 | 21.80 | 16.44 | Mutated |
| *SMARCB1* | 0.64 | 13.30 | 16.52 | Mutated |
| *FAT1* | 0.70 | 14.62 | 16.52 | Mutated |
| *NF2* | 0.70 | 19.16 | 16.46 | Mutated |
| *TP53* | 0.71 | 16.93 | 16.46 | Mutated |

Supplementary table 5. Association of genomic alterations and stroma texture in samples without normal tissue.

| **genes** | **pvalue** | **Class 1 median** | **Class 2 median** | **Class 1 value** |
| --- | --- | --- | --- | --- |
| *SETD2* | 0.031 | 8.65 | 12.38 | Mutated |
| *SMARCB1* | 0.085 | 0.00 | 11.58 | Mutated |
| *STAG2* | 0.10 | 17.19 | 11.52 | Mutated |
| *BAP1* | 0.16 | 9.94 | 11.90 | Mutated |
| *NF2* | 0.17 | 1.53 | 11.58 | Mutated |
| Mutations total | 0.22 | 11.21 | 12.07 | High burden |
| *PTEN* | 0.32 | 13.49 | 11.57 | Mutated |
| Ploidy | 0.43 | 11.77 | 11.71 | Diploid |
| *TP53* | 0.44 | 9.94 | 11.71 | Mutated |
| *FAT1* | 0.45 | 15.09 | 11.57 | Mutated |
| *PBRM1* | 0.57 | 11.83 | 11.53 | Mutated |
| *MTOR* | 0.70 | 10.17 | 11.64 | Mutated |
| *KDM6A* | 0.71 | 9.01 | 11.58 | Mutated |
| *PIK3CA* | 0.78 | 12.04 | 11.57 | Mutated |
| *KDM5C* | 0.84 | 12.31 | 11.57 | Mutated |
| *VHL* | 0.87 | 11.57 | 11.58 | Mutated |
| *NFE2L2* | 0.88 | 13.5 | 11.57 | Mutated |
