# Supplementary material for: Computational textural mapping harmonises sampling variation and reveals multidimensional histopathological fingerprints
Source: Br J Cancer. 2023 Jun 30;129(4):683–95. doi: 10.1038/s41416-023-02329-4 (PMC10421901; doi:10.1038/s41416-023-02329-4)
Supplement: Supplementary file 2 — Supplemental figures [file 41416_2023_2329_MOESM2_ESM.pptx]

## Slide 1
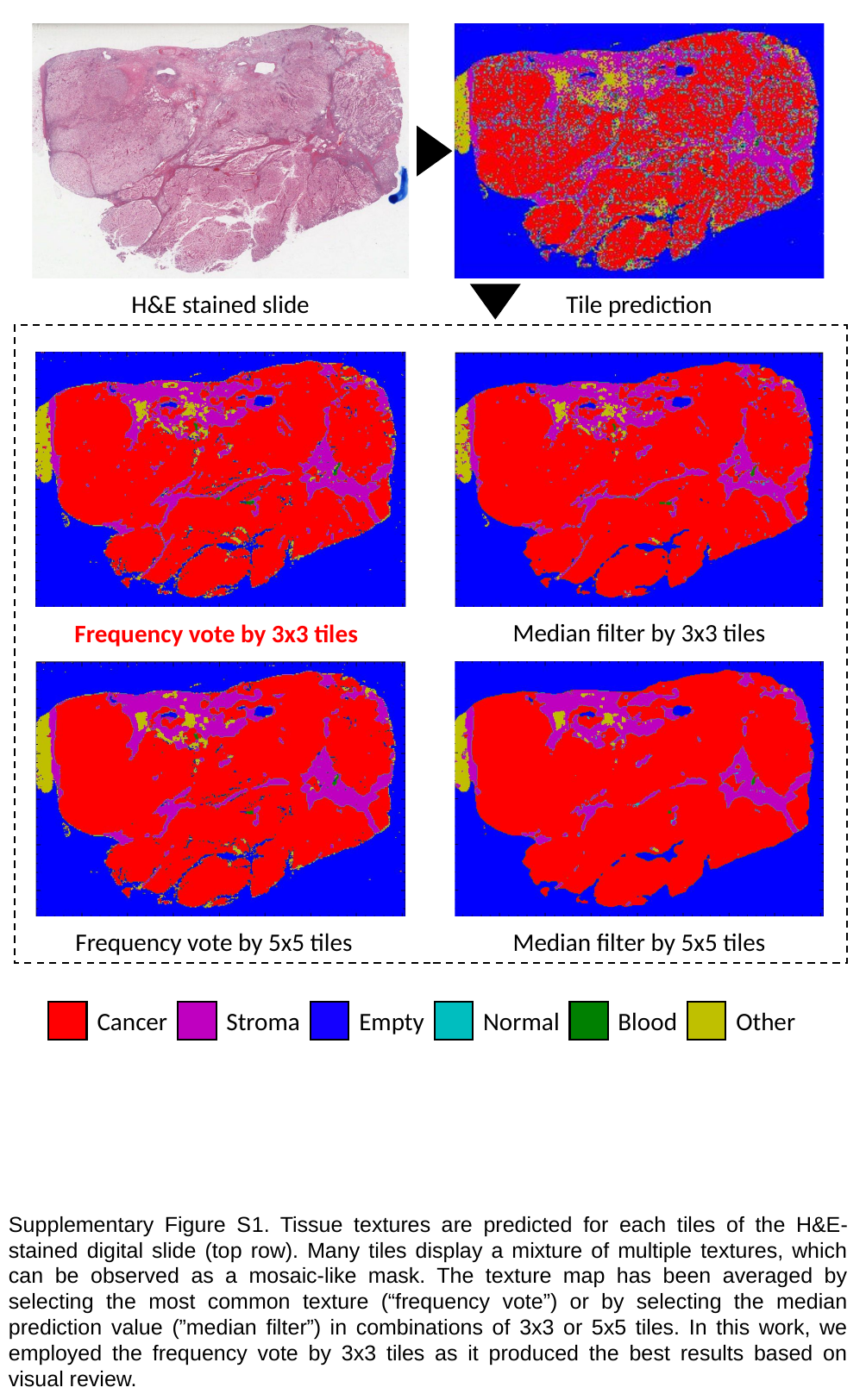

H&E stained slide
Tile prediction
Median filter by 3x3 tiles
Frequency vote by 3x3 tiles
Frequency vote by 5x5 tiles
Median filter by 5x5 tiles
Cancer
Stroma
Empty
Normal
Blood
Other
Supplementary Figure S1. Tissue textures are predicted for each tiles of the H&E-stained digital slide (top row). Many tiles display a mixture of multiple textures, which can be observed as a mosaic-like mask. The texture map has been averaged by selecting the most common texture (“frequency vote”) or by selecting the median prediction value (”median filter”) in combinations of 3x3 or 5x5 tiles. In this work, we employed the frequency vote by 3x3 tiles as it produced the best results based on visual review.

## Slide 2
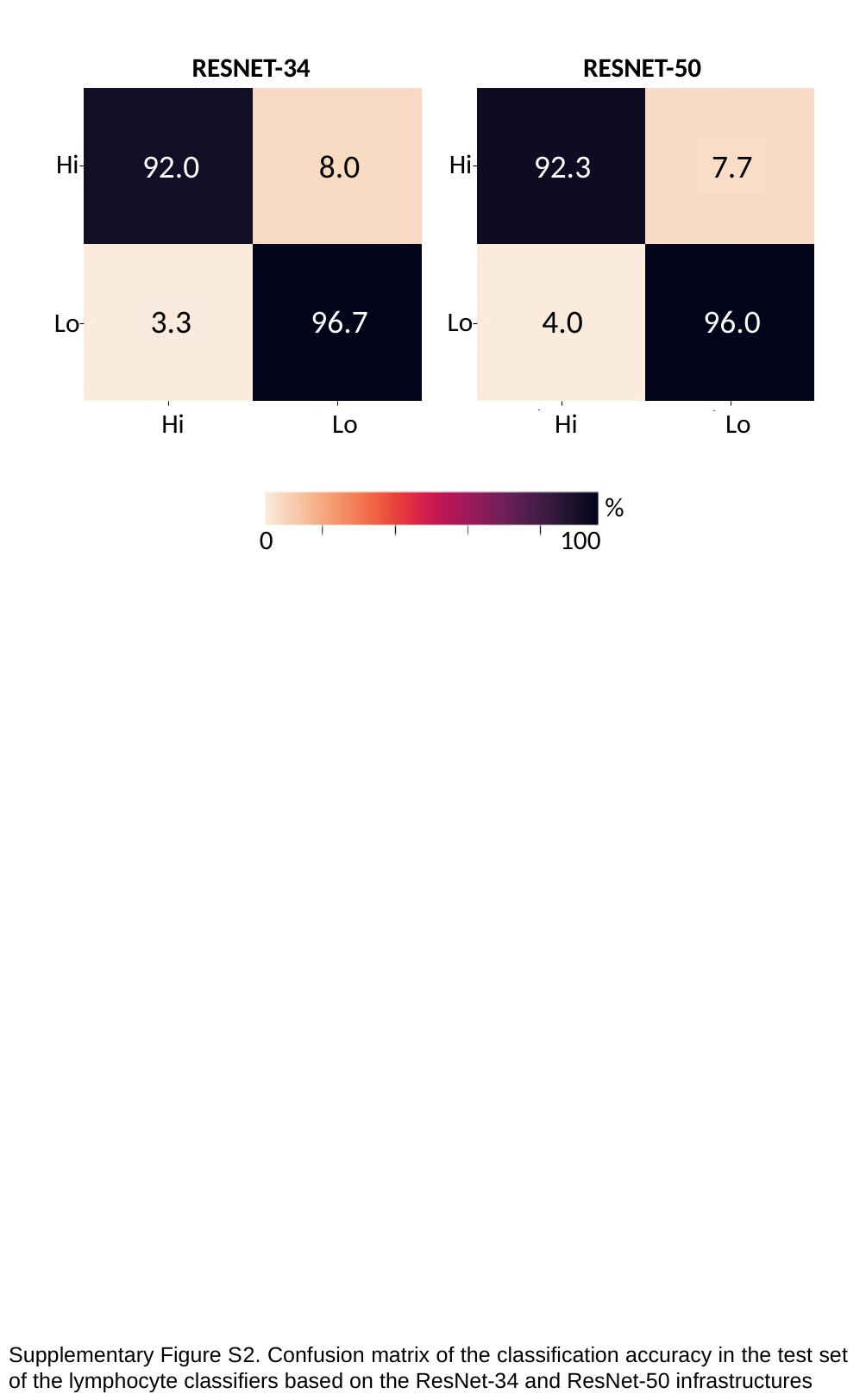

RESNET-34
RESNET-50
Hi
Lo
Hi
Lo
Hi
Lo
Hi
Lo
92.0
8.0
92.3
7.7
3.3
96.7
4.0
96.0
%
0
100
Supplementary Figure S2. Confusion matrix of the classification accuracy in the test set of the lymphocyte classifiers based on the ResNet-34 and ResNet-50 infrastructures

## Slide 3
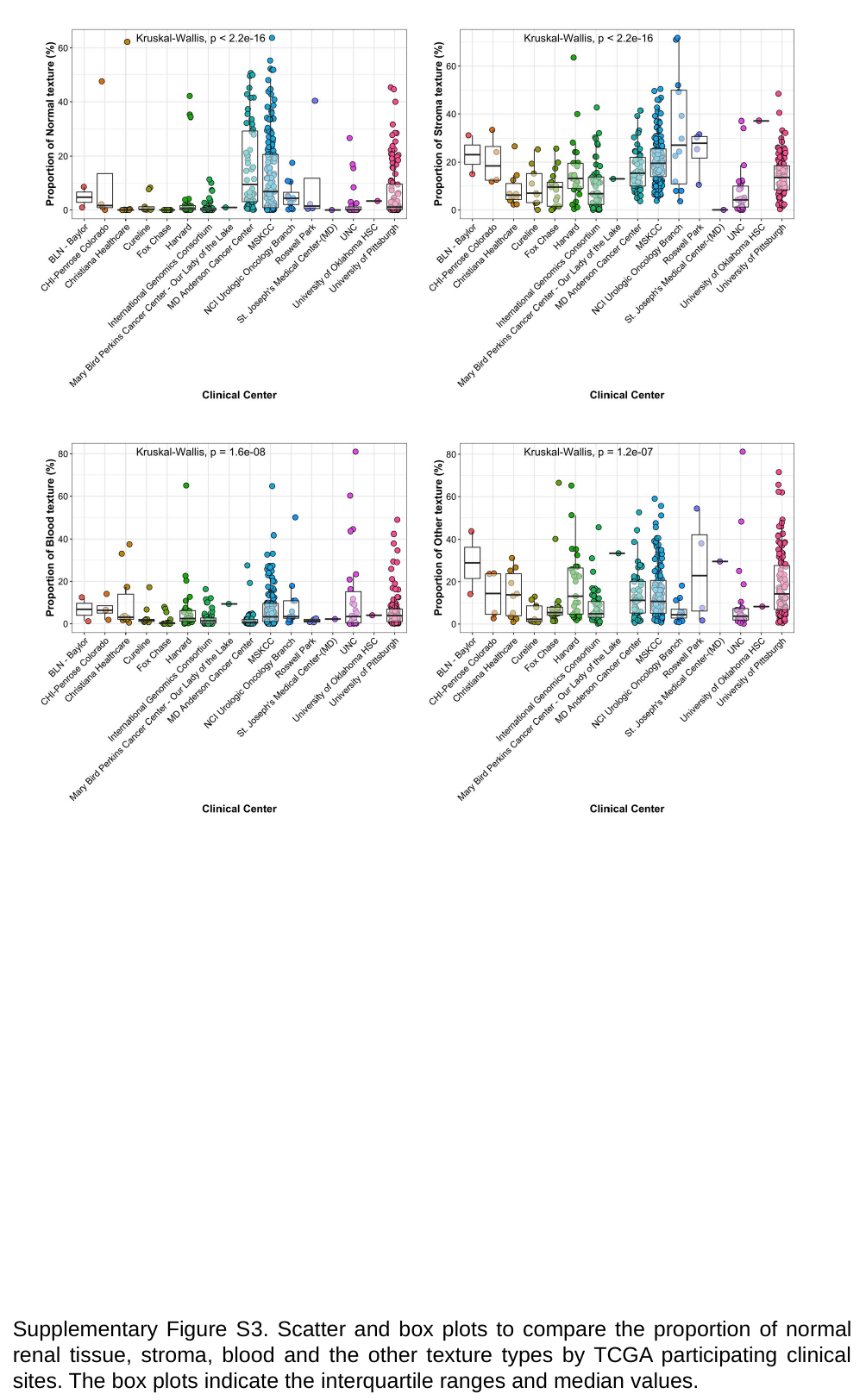

Supplementary Figure S3. Scatter and box plots to compare the proportion of normal renal tissue, stroma, blood and the other texture types by TCGA participating clinical sites. The box plots indicate the interquartile ranges and median values.

## Slide 4
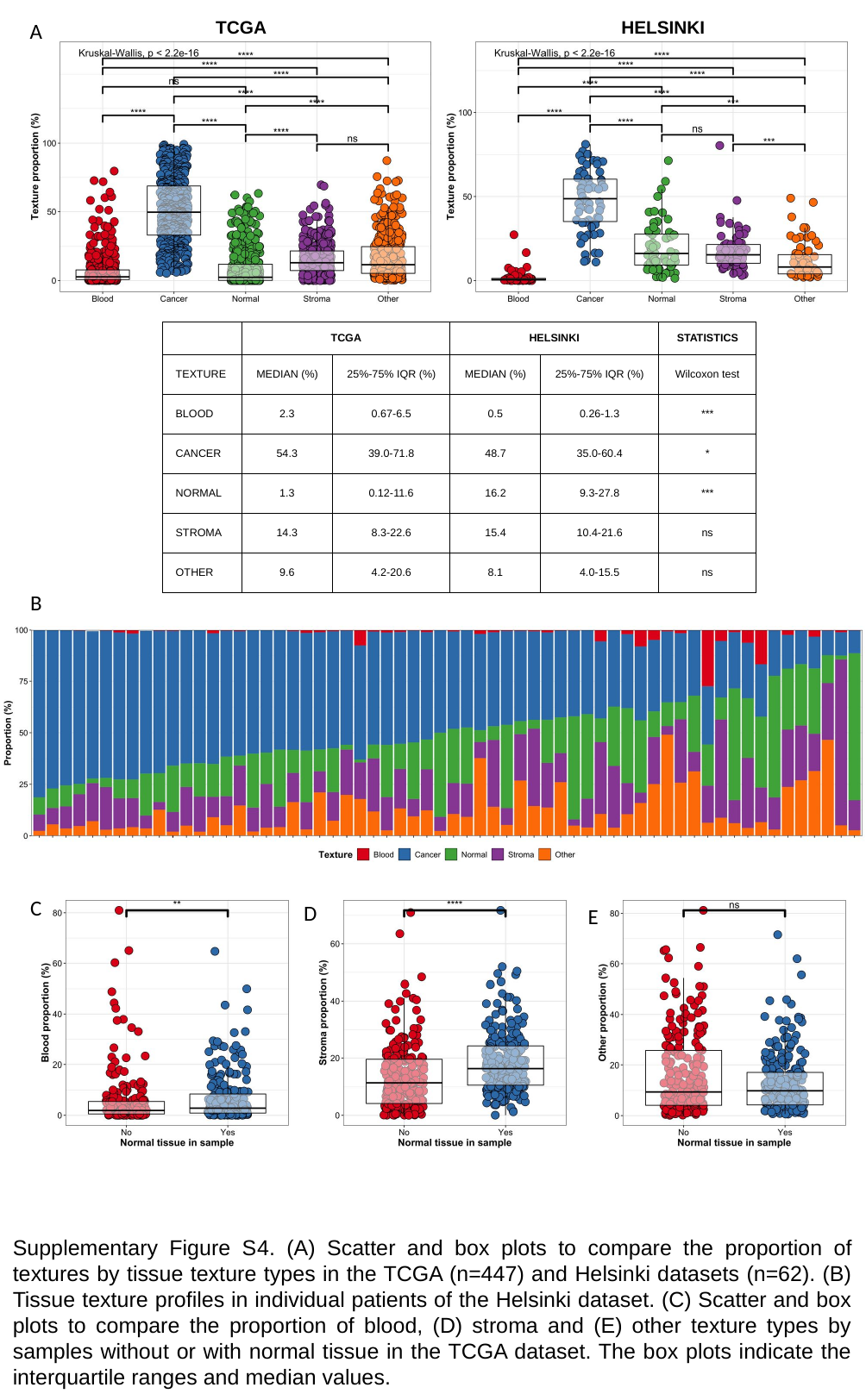

TCGA
HELSINKI
A
| | TCGA | | HELSINKI | | STATISTICS |
| --- | --- | --- | --- | --- | --- |
| TEXTURE | MEDIAN (%) | 25%-75% IQR (%) | MEDIAN (%) | 25%-75% IQR (%) | Wilcoxon test |
| BLOOD | 2.3 | 0.67-6.5 | 0.5 | 0.26-1.3 | \*\*\* |
| CANCER | 54.3 | 39.0-71.8 | 48.7 | 35.0-60.4 | \* |
| NORMAL | 1.3 | 0.12-11.6 | 16.2 | 9.3-27.8 | \*\*\* |
| STROMA | 14.3 | 8.3-22.6 | 15.4 | 10.4-21.6 | ns |
| OTHER | 9.6 | 4.2-20.6 | 8.1 | 4.0-15.5 | ns |
B
C
D
E
Supplementary Figure S4. (A) Scatter and box plots to compare the proportion of textures by tissue texture types in the TCGA (n=447) and Helsinki datasets (n=62). (B) Tissue texture profiles in individual patients of the Helsinki dataset. (C) Scatter and box plots to compare the proportion of blood, (D) stroma and (E) other texture types by samples without or with normal tissue in the TCGA dataset. The box plots indicate the interquartile ranges and median values.

## Slide 5
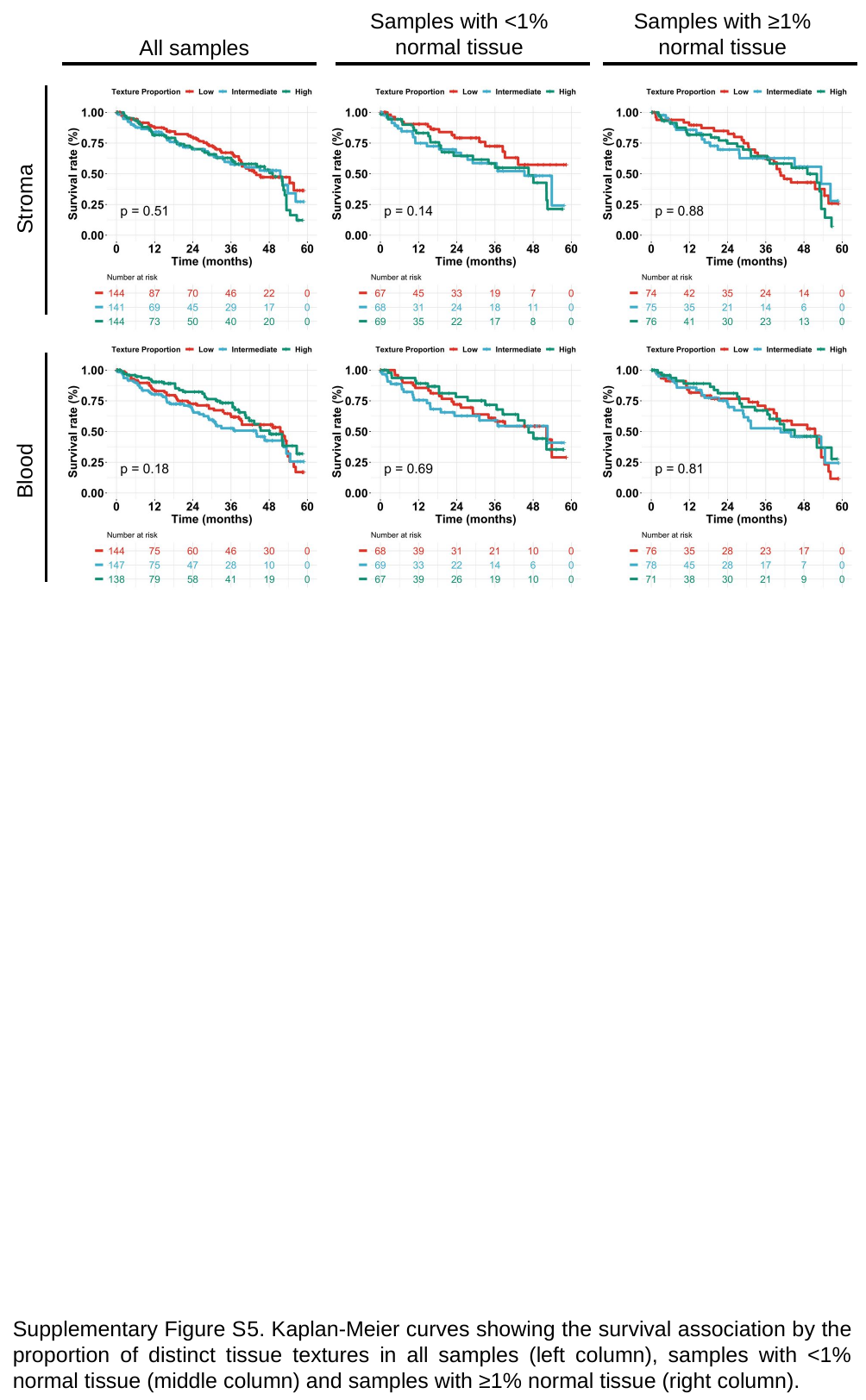

Samples with <1% normal tissue
Samples with ≥1% normal tissue
All samples
Stroma
Blood
Supplementary Figure S5. Kaplan-Meier curves showing the survival association by the proportion of distinct tissue textures in all samples (left column), samples with <1% normal tissue (middle column) and samples with ≥1% normal tissue (right column).

## Slide 6
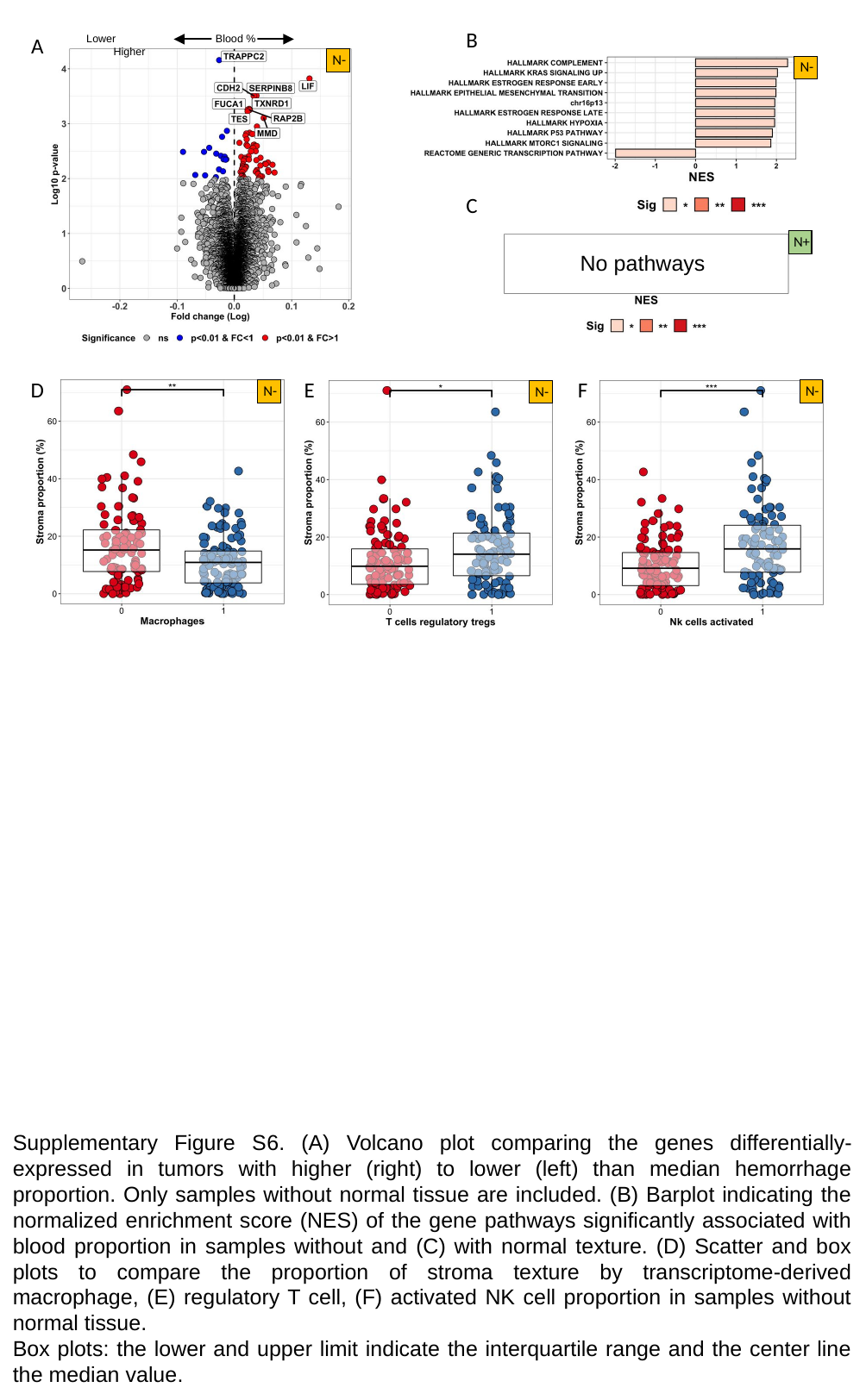

B
A
Blood %
Lower	 Higher
N-
N-
C
N+
No pathways
D
E
F
N-
N-
N-
Supplementary Figure S6. (A) Volcano plot comparing the genes differentially-expressed in tumors with higher (right) to lower (left) than median hemorrhage proportion. Only samples without normal tissue are included. (B) Barplot indicating the normalized enrichment score (NES) of the gene pathways significantly associated with blood proportion in samples without and (C) with normal texture. (D) Scatter and box plots to compare the proportion of stroma texture by transcriptome-derived macrophage, (E) regulatory T cell, (F) activated NK cell proportion in samples without normal tissue.
Box plots: the lower and upper limit indicate the interquartile range and the center line the median value.

## Slide 7
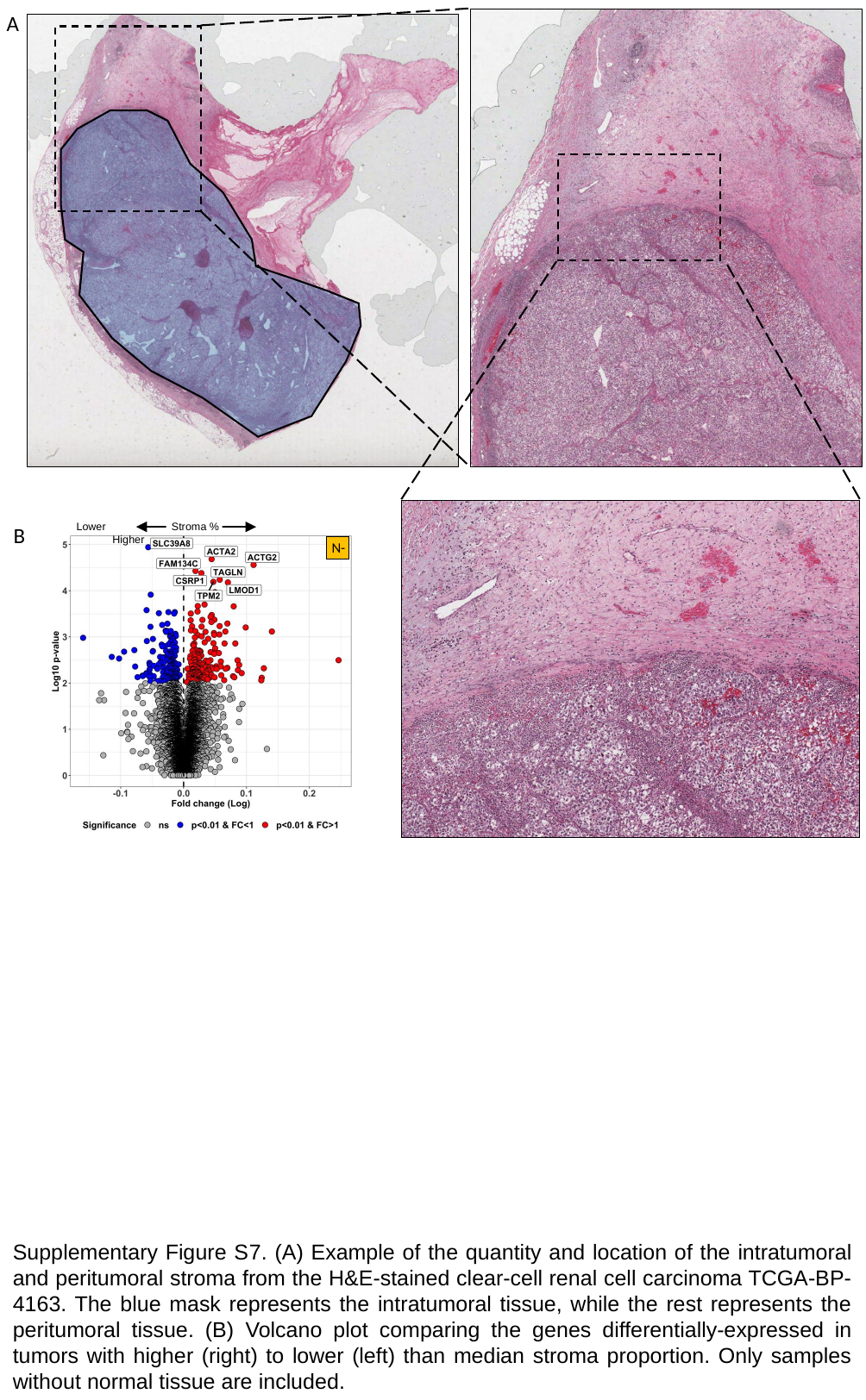

A
Stroma %
Lower	 Higher
N-
B
Supplementary Figure S7. (A) Example of the quantity and location of the intratumoral and peritumoral stroma from the H&E-stained clear-cell renal cell carcinoma TCGA-BP-4163. The blue mask represents the intratumoral tissue, while the rest represents the peritumoral tissue. (B) Volcano plot comparing the genes differentially-expressed in tumors with higher (right) to lower (left) than median stroma proportion. Only samples without normal tissue are included.

## Slide 8
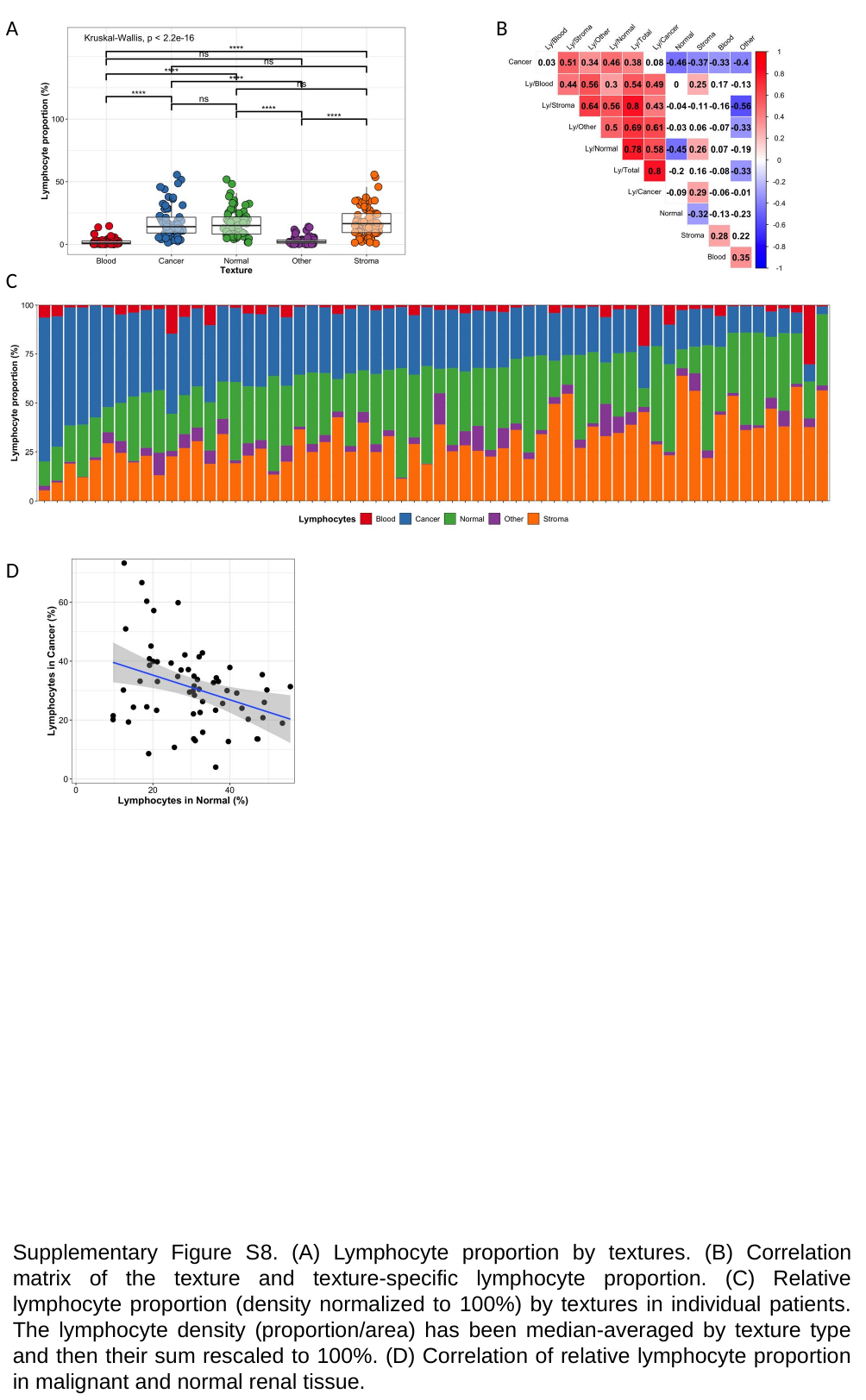

A
B
C
D
Supplementary Figure S8. (A) Lymphocyte proportion by textures. (B) Correlation matrix of the texture and texture-specific lymphocyte proportion. (C) Relative lymphocyte proportion (density normalized to 100%) by textures in individual patients. The lymphocyte density (proportion/area) has been median-averaged by texture type and then their sum rescaled to 100%. (D) Correlation of relative lymphocyte proportion in malignant and normal renal tissue.

## Slide 9
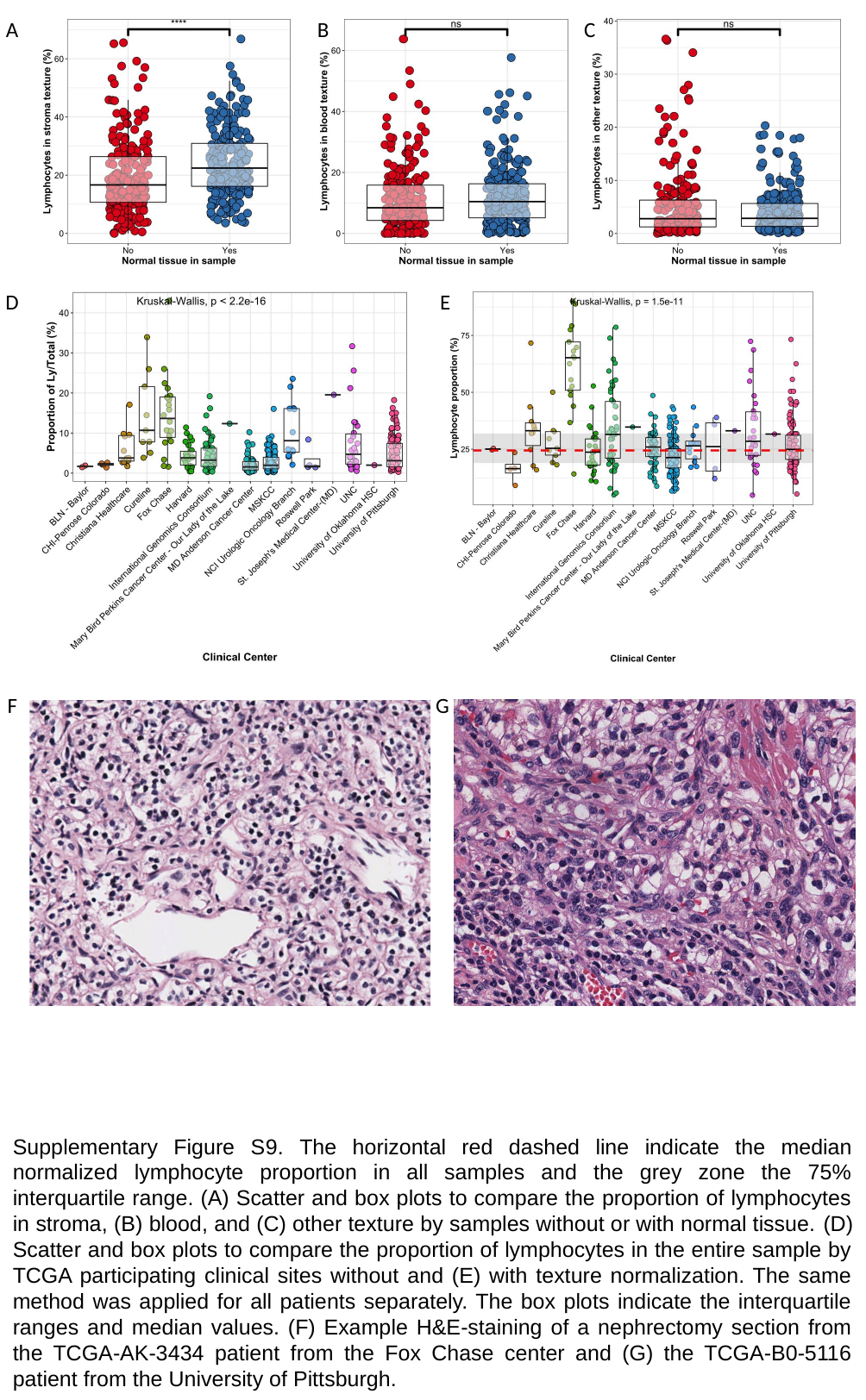

A
B
C
D
E
F
G
Supplementary Figure S9. The horizontal red dashed line indicate the median normalized lymphocyte proportion in all samples and the grey zone the 75% interquartile range. (A) Scatter and box plots to compare the proportion of lymphocytes in stroma, (B) blood, and (C) other texture by samples without or with normal tissue. (D) Scatter and box plots to compare the proportion of lymphocytes in the entire sample by TCGA participating clinical sites without and (E) with texture normalization. The same method was applied for all patients separately. The box plots indicate the interquartile ranges and median values. (F) Example H&E-staining of a nephrectomy section from the TCGA-AK-3434 patient from the Fox Chase center and (G) the TCGA-B0-5116 patient from the University of Pittsburgh.

## Slide 10
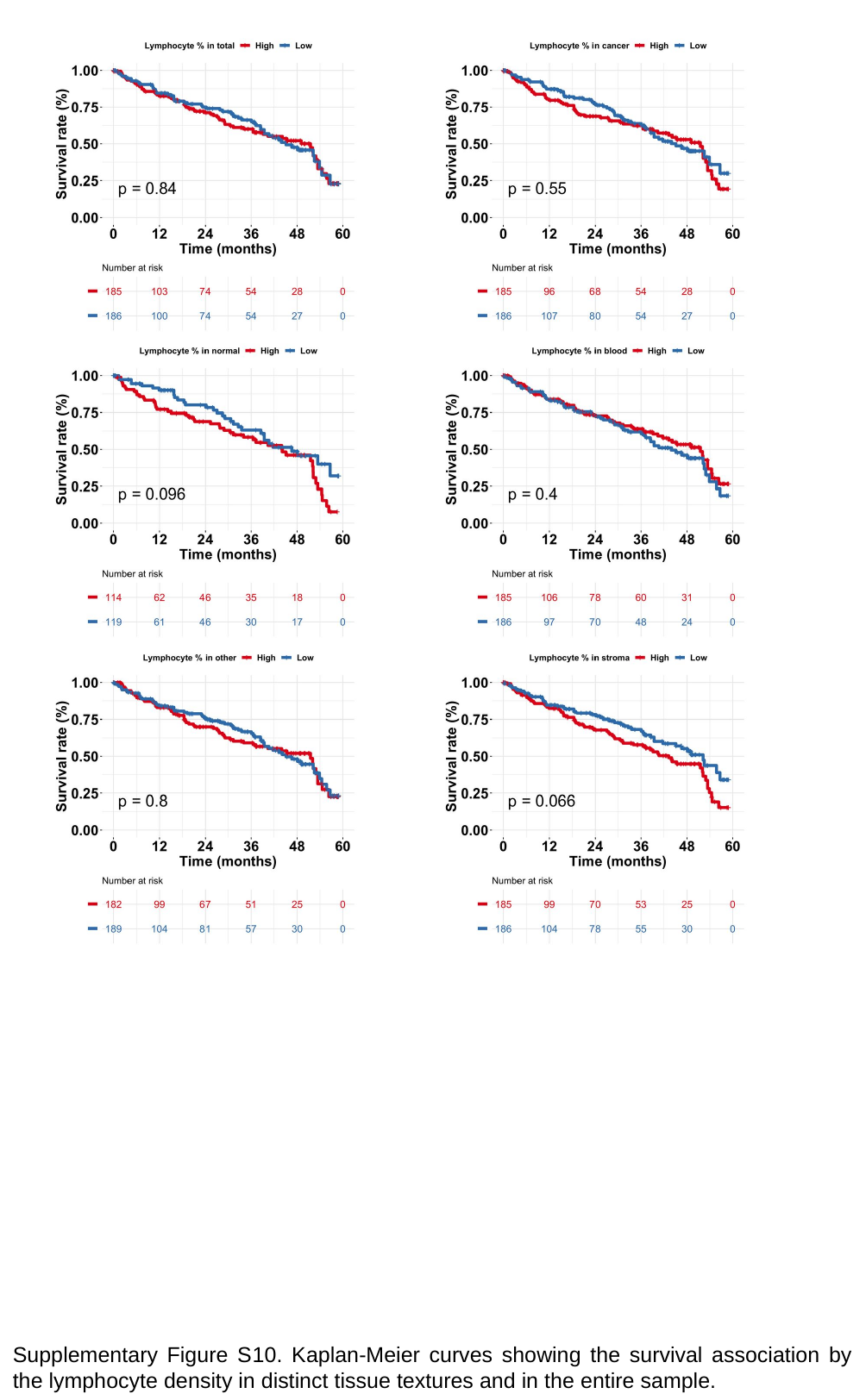

Supplementary Figure S10. Kaplan-Meier curves showing the survival association by the lymphocyte density in distinct tissue textures and in the entire sample.

## Slide 11
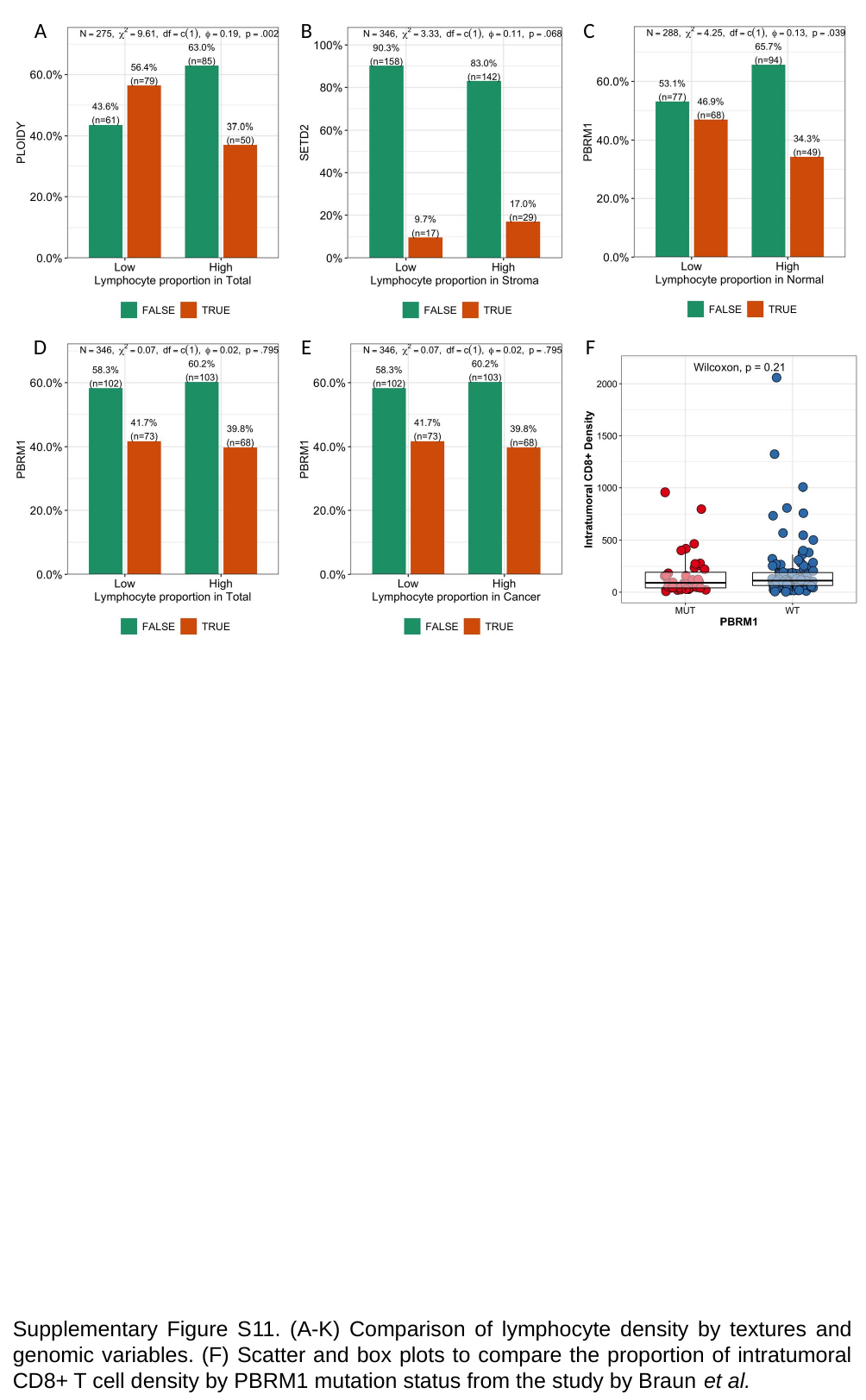

A
B
C
D
E
F
Supplementary Figure S11. (A-K) Comparison of lymphocyte density by textures and genomic variables. (F) Scatter and box plots to compare the proportion of intratumoral CD8+ T cell density by PBRM1 mutation status from the study by Braun et al.

## Slide 12
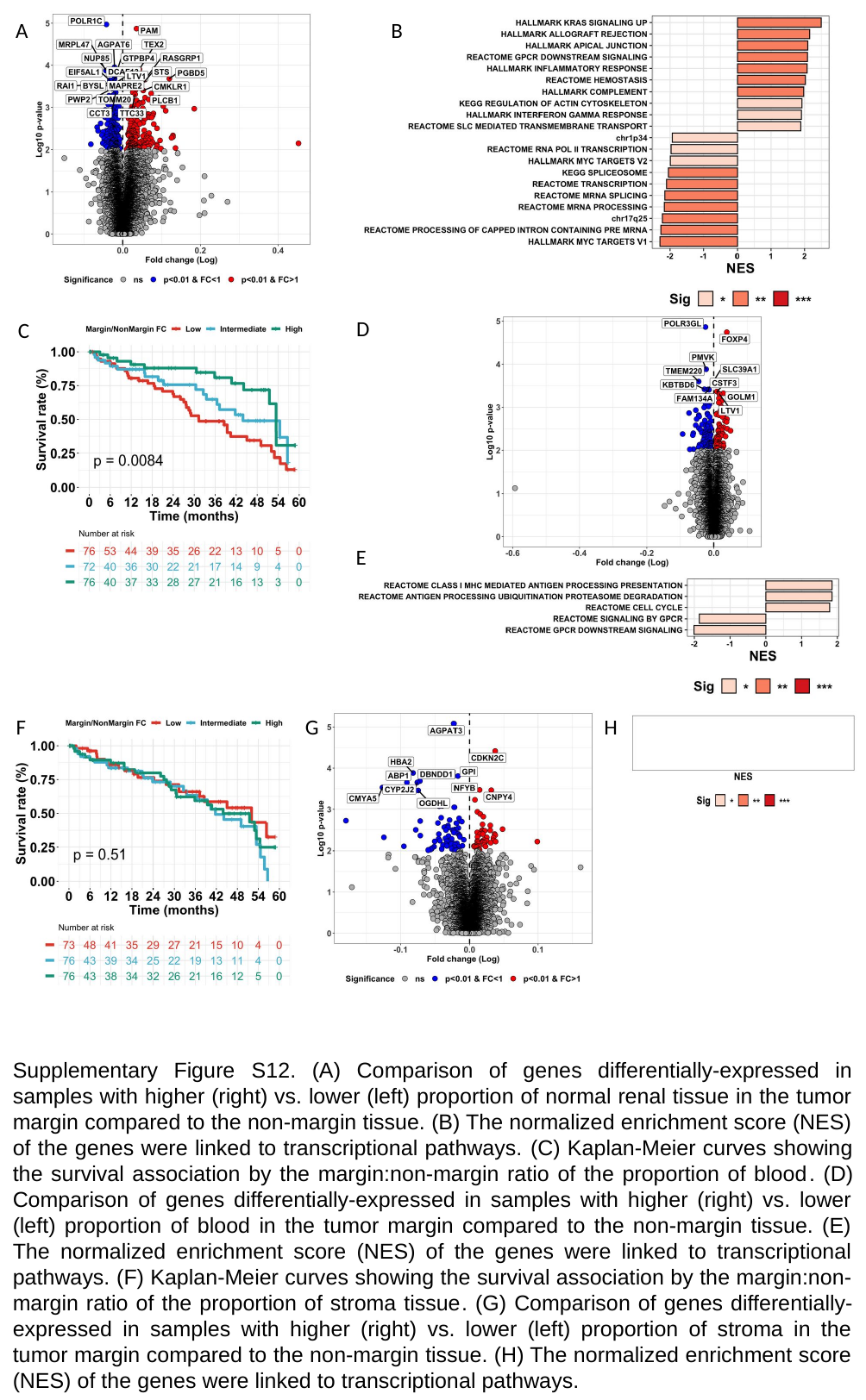

A
B
D
C
E
F
G
H
Supplementary Figure S12. (A) Comparison of genes differentially-expressed in samples with higher (right) vs. lower (left) proportion of normal renal tissue in the tumor margin compared to the non-margin tissue. (B) The normalized enrichment score (NES) of the genes were linked to transcriptional pathways. (C) Kaplan-Meier curves showing the survival association by the margin:non-margin ratio of the proportion of blood. (D) Comparison of genes differentially-expressed in samples with higher (right) vs. lower (left) proportion of blood in the tumor margin compared to the non-margin tissue. (E) The normalized enrichment score (NES) of the genes were linked to transcriptional pathways. (F) Kaplan-Meier curves showing the survival association by the margin:non-margin ratio of the proportion of stroma tissue. (G) Comparison of genes differentially-expressed in samples with higher (right) vs. lower (left) proportion of stroma in the tumor margin compared to the non-margin tissue. (H) The normalized enrichment score (NES) of the genes were linked to transcriptional pathways.

## Slide 13
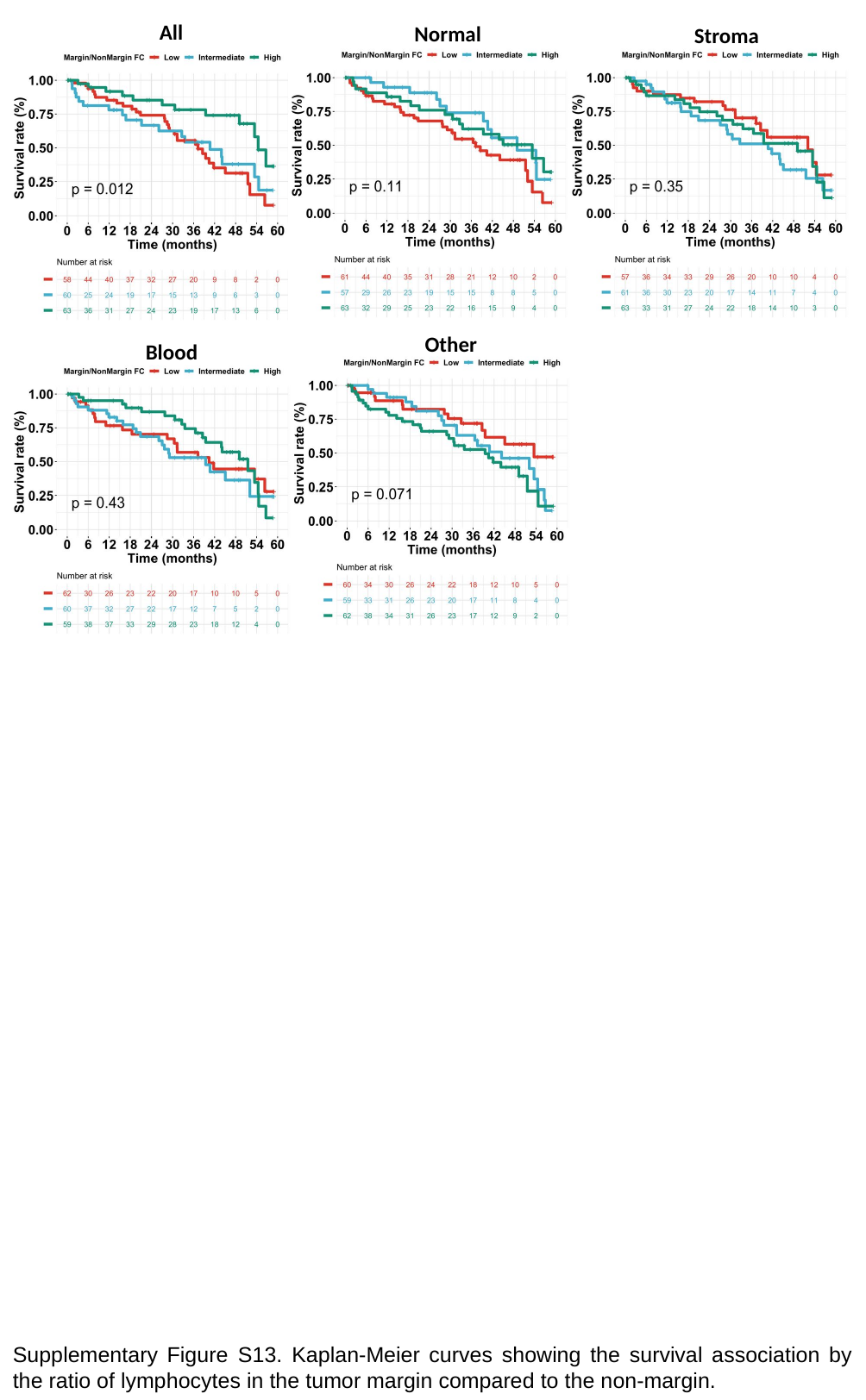

All
Normal
Stroma
Other
Blood
Supplementary Figure S13. Kaplan-Meier curves showing the survival association by the ratio of lymphocytes in the tumor margin compared to the non-margin.

## Slide 14
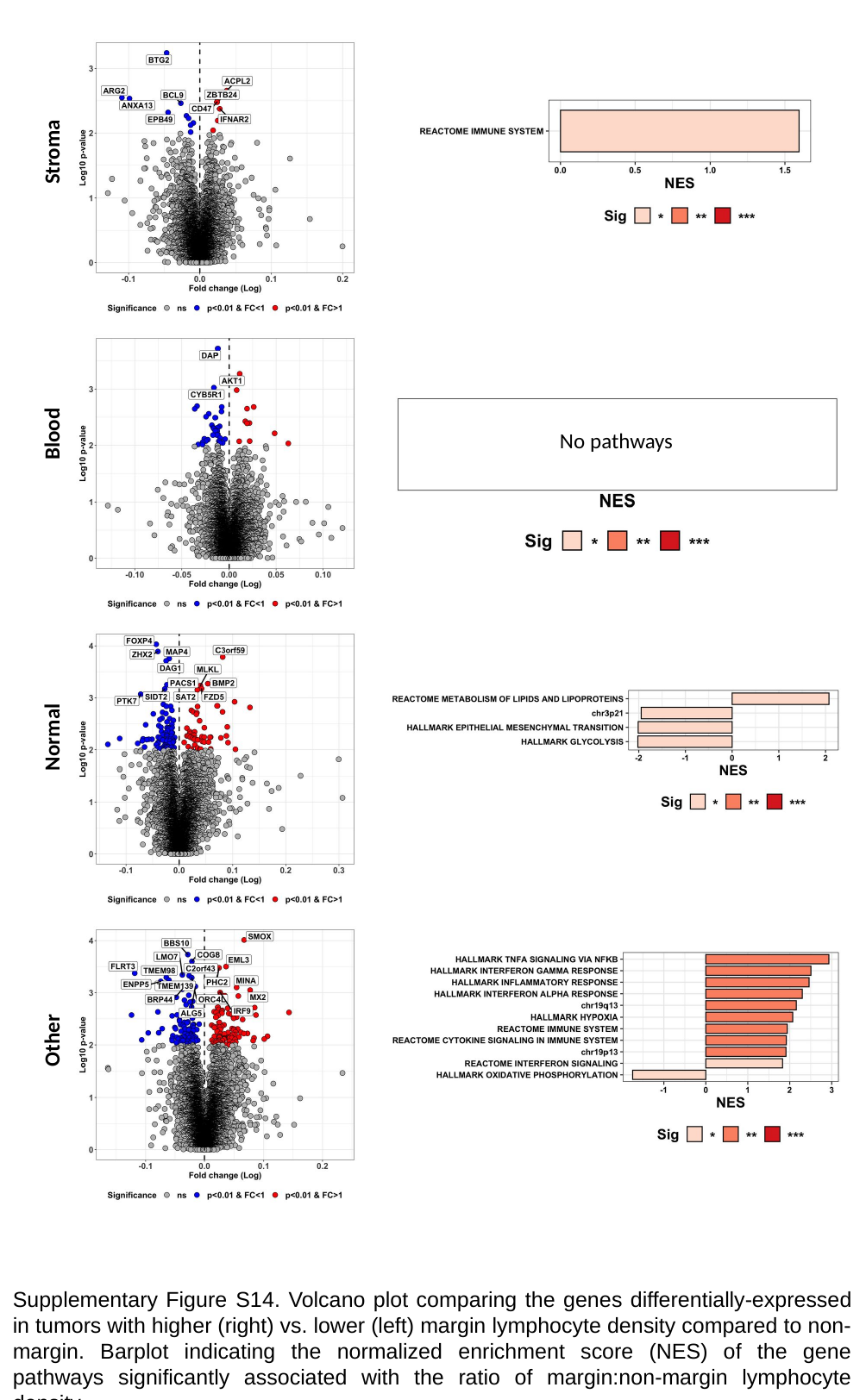

Stroma
Blood
No pathways
Normal
Other
Supplementary Figure S14. Volcano plot comparing the genes differentially-expressed in tumors with higher (right) vs. lower (left) margin lymphocyte density compared to non-margin. Barplot indicating the normalized enrichment score (NES) of the gene pathways significantly associated with the ratio of margin:non-margin lymphocyte density.

## Slide 15
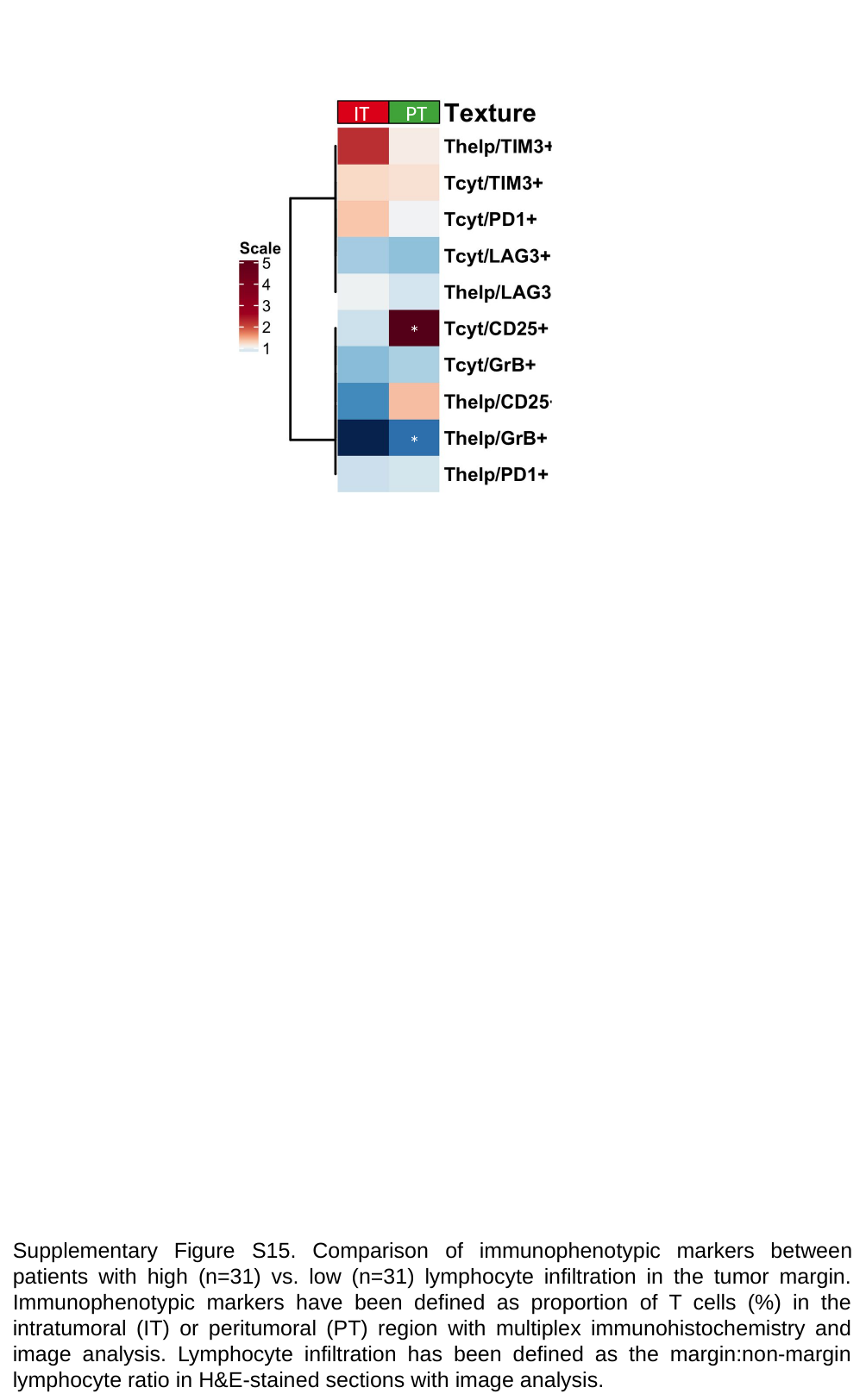

IT
PT
*
*
Supplementary Figure S15. Comparison of immunophenotypic markers between patients with high (n=31) vs. low (n=31) lymphocyte infiltration in the tumor margin. Immunophenotypic markers have been defined as proportion of T cells (%) in the intratumoral (IT) or peritumoral (PT) region with multiplex immunohistochemistry and image analysis. Lymphocyte infiltration has been defined as the margin:non-margin lymphocyte ratio in H&E-stained sections with image analysis.
